# Genetic Variance in Heparan Sulfation Is Associated With Salt Sensitivity

**DOI:** 10.1161/HYPERTENSIONAHA.124.23421

**Published:** 2024-09-09

**Authors:** Jetta J. Oppelaar, Bart Ferwerda, Mohamed A. Romman, Ghazalah N. Sahebdin, Aeilko H. Zwinderman, Henrike Galenkamp, S. Matthijs Boekholdt, Bert-Jan H. van den Born, Rik H.G. Olde Engberink, Liffert Vogt

**Affiliations:** Department of Internal Medicine, Section of Nephrology (J.J.O., M.A.R., G.N.S., R.H.G.O.E., L.V.); Department of Clinical Epidemiology and Biostatistics (B.F., A.H.Z.); Department of Cardiology (S.M.B.); Department of Public and Occupational Health, Amsterdam Public Health (H.G., B.-J.H.B.); Department of Internal Medicine, Section of Vascular Medicine, Amsterdam University Medical Center location University of Amsterdam, Meibergdreef 9, the Netherlands (B.-J.H.B.).; Amsterdam Cardiovascular Sciences, the Netherlands (J.J.O., R.H.G.O.E., L.V., S.M.B., B.-J.H.B.).

**Keywords:** blood pressure, glycosaminoglycans, salt tolerance, sodium, UK Biobank

## Abstract

**BACKGROUND:**

High heritability of salt sensitivity suggests an essential role for genetics in the relationship between sodium intake and blood pressure (BP). The role of glycosaminoglycan genes, which are crucial for salinity tolerance, remains to be elucidated.

**METHODS:**

Interactions between 54 126 variants in 130 glycosaminoglycan genes and daily sodium excretion on BP were explored in 20 420 EPIC-Norfolk (European Prospective Investigation Into Cancer in Norfolk) subjects. The UK Biobank (n=414 132) and the multiethnic HELIUS study (Healthy Life in an Urban Setting; n=2239) were used for validation. Afterward, the urinary glycosaminoglycan composition was studied in HELIUS participants (n=57) stratified by genotype and upon dietary sodium loading in a time-controlled crossover intervention study (n=12).

**RESULTS:**

rs2892799 in *NDST3* (heparan sulfate N-deacetylase/N-sulfotransferase 3) showed the strongest interaction with sodium on mean arterial pressure (false discovery rate 0.03), with higher mean arterial pressure for the C allele in high sodium conditions. Also, rs9654628 in *HS3ST5* (heparan sulfate-glucosamine 3-sulfotransferase 5) showed an interaction with sodium on systolic BP (false discovery rate 0.03). These interactions were multiethnically validated. Stratifying for the rs2892799 genotype showed higher urinary expression of N-sulfated heparan sulfate epitope D0S0 for the T allele. Conversely, upon dietary sodium loading, urinary D0S0 expression was higher in participants with stable BP after sodium loading, and sodium-induced effects on this epitope were opposite in individuals with and without BP response to sodium.

**CONCLUSIONS:**

The C allele of rs2892799 in *NDST3* exhibits higher BP in high sodium conditions when compared with low sodium conditions, whereas no differences were detected for the T allele. Concomitantly, both alleles demonstrate distinct expressions of D0S0, which, in turn, correlates with sodium-mediated BP elevation. These findings underscore the potential significance of genetic glycosaminoglycan variation in human BP regulation.

**NOVELTY AND RELEVANCE**

**What Is New?**

- Genetic variants in *NDST3* (heparan sulfate N-deacetylase/N-sulfotransferase 3) and *HS3ST5* (heparan sulfate-glucosamine 3-sulfotransferase 5) genes show interaction with daily sodium consumption on blood pressure.
- No salt-sensitive blood pressure response could be observed in individuals with the T allele of rs2892799 in *NDST3*. Concomitantly, the T allele is associated with higher urinary expression of the N-sulfated heparan sulfate epitope D0S0. Irrespective of genotype, this epitope significantly differed between salt-sensitive and salt-resistant individuals after dietary sodium loading.

**What Is Relevant?**

- Genetic variation in metabolic glycosaminoglycan adaptation enzymes is for the first time linked to salt sensitivity of blood pressure in humans.

**Clinical/Pathophysiological Implications?**

- Glycosaminoglycans are dynamic polysaccharides that play an important role in the link between sodium consumption and blood pressure regulation.
- In line with earlier observations from controlled clinical trials, targeting glycosaminoglycan metabolic adaptation may prove to negate the adverse long-term effects of high sodium consumption.

Glycosaminoglycans are complex polysaccharides constituting a significant portion of the extracellular matrix.^1^ Glycosaminoglycans can be categorized into unsulfated hyaluronic acid and 4 groups of sulfated glycosaminoglycans, including heparin/heparan sulfate, chondroitin sulfate/dermatan sulfate, and keratin sulfate.^2^ The core disaccharide units of sulfated glycosaminoglycans can be modified by the spatiotemporally controlled expression of sulfotransferase and epimerase enzymes.^3^ Because of secondary modifications, glycosaminoglycan biosynthesis is highly heterogenic, which is essential for facilitating a myriad of biological functions.^4,5^ Sulfated glycosaminoglycans and associated enzymes are present in both vertebrates and invertebrates.^6^ The sequence homology between the coding regions of different species can show up to 98% homology.^7^ Together, this suggests a preserved function across the entire animal kingdom. Organisms in high-salt environments, such as sharks, crabs, squids, and mollusks, exhibit complex and highly sulfated glycosaminoglycan structures.^8–11^ Additionally, the level of sulfation in glycosaminoglycans increases with the salinity of the organism’s habitat, suggesting a significant role for glycosaminoglycan sulfation in adapting to high-salt conditions.^12^

In humans, dietary patterns have transitioned from primarily consuming fresh foods to processed foods rich in sodium. Strong and consistent evidence within and across populations indicates high sodium intake is a significant risk factor for high blood pressure (BP).^13–15^ The extent to which sodium intake affects BP varies considerably within and across populations, discriminating salt-sensitive subjects from salt-resistant ones.^16^ Evidence from human and animal studies has demonstrated that sodium is not exclusively restricted to the extracellular volume and can accumulate in tissues without commensurate changes in body water content.^17–19^ In both animal and human studies, increased tissue salinity after high sodium intake coincides with increased glycosaminoglycan synthesis, polymerization, and sulfation.^20,21^ About the role of glycosaminoglycan sulfation in adaptation to the salinity of the environment in nonhuman organisms, we hypothesized that genetic disturbances in sodium handling by glycosaminoglycans will bear consequences for the salt sensitivity of BP. Therefore, we performed a candidate gene study in Europeans from the EPIC-Norfolk (European Prospective Investigation Into Cancer in Norfolk) and UK Biobank (UKBB) cohorts to investigate the interaction between single-nucleotide polymorphisms (SNPs) in genes involved in glycosaminoglycan and proteoglycan biosynthesis and sodium intake on BP. Considering the high prevalence of salt sensitivity and high heritability in non-European populations^16^ and the current ancestry imbalance in genetic salt-sensitivity studies, we performed validation in the multiethnic HELIUS (Healthy Life in an Urban Setting) cohort. glycosaminoglycan expression patterns were studied among carriers of glycosaminoglycan gene variants and salt-sensitive and salt-resistant individuals.

## METHODS

### Data Availablility

The data that support the findings of this study are available from the corresponding author upon reasonable request. Besides the sections below, detailed methods are available in the Supplemental Material.

### Study Population

For this candidate gene approach, we studied the interaction between sodium intake and SNPs in glycosaminoglycan metabolism-associated genes in individuals from the EPIC-Norfolk and UKBB cohorts. EPIC-Norfolk was used as the discovery cohort. Details of both cohorts have been previously described elsewhere.^22,23^ Briefly, EPIC-Norfolk is a prospective population study in 25 639 men and women aged 40 to 79 years who were randomly recruited from general practices in Norfolk, United Kingdom. Analysis of baseline data showed that participants were comparable with the national population regarding BP and other cardiovascular risk factors such as kidney function and smoking.^15^ The Norfolk Health District Ethics Committee approved the EPIC-Norfolk study, and the EPIC-Norfolk Management Committee approved our analysis (ENDR028_2020). UKBB is a population-based cohort study of individuals recruited from 22 rural and urban recruitment centers in the United Kingdom. It includes clinical, demographic, and genotypic information of 500 000 individuals aged 40 to 69 years at the time of inclusion. The National Research Ethics Service Committee approved UKBB protocols, and the analyses in this study were conducted under the approved UKBB application 75038. In both cohorts, we selected all individuals of European ancestry with complete data for BP, body mass index (BMI), and urine levels of sodium, potassium, and creatinine. UKBB participants who withdrew their consent during follow-up were excluded.

### BP and Urinary Sodium and Potassium Measurements

In EPIC-Norfolk, duplicate BP measurements were performed using an automatic noninvasive oscillometric BP measuring device (Datascope Accutorr Plus; Datascope Medical) after the subject had been seated for 5 minutes.^15^ UKBB measured seated BP after 5 slow breaths in a relaxed fashion (Omron 705-IT; Omron Healthcare Inc).^24^ The mean of 2 sequential readings was used for analysis in both cohorts. Mean arterial pressure (MAP) was calculated as $\;\left(\frac{1}{3}*\left(systolic\;\;\;BP-diastolic\;\;\;BP\right)+diastolic\;\;\;BP\right)$. All participants from both cohorts collected a random casual (spot) urine sample, which was analyzed for sodium, potassium, and creatinine concentrations. Spot urine sodium excretion was used to estimate daily sodium intake by calculating 24-hour sodium excretion using the Kawasaki formula^25^ and the Tanaka formula^26^ for validation (Appendix S1). After applying the Kawasaki formula, outliers for sodium excretion were excluded if values were 3× the interquartile range below or above the first and third quartiles, respectively.

### Selection of Relevant SNPs

Reactome was used to identify genes involved in synthesizing and modifying keratin sulfate, heparan sulfate, chondroitin sulfate, and hyaluronic acid.^27^ With PLINK2 software,^28,29^ all SNPs located at autosomal chromosomes within the genomic regions of these genes or within ±10 kbp of these genomic regions were selected for our analysis. Literature was searched for the identification of additional relevant genes in glycosaminoglycan homeostasis.

### Genotyping, Genomic Imputation, and Quality Control

EPIC-Norfolk and most (≈90%) UKBB samples were genotyped on the UKBB Axiom Array. A small part (≈10%) of the UKBB samples was genotyped on Affymetrix UK BiLEVE Axiom Array, which shared 95% coverage with the UKBB Axiom Array.^30^ Imputation in EPIC-Norfolk and UKBB was performed centrally into the same reference panels. The Haplotype Reference Consortium reference panel was used as the primary reference; SNPs that did not occur on this panel were imputed with the UK10K and 1000G reference panels. Our analysis was conducted with version 3 of the UKBB imputed data for which the UKBB centrally applied quality control filters for imputation.^31^ In EPIC-Norfolk, we selected SNPs based on 3 thresholds for quality control: (1) minor allele frequency ≥0.01, (2) INFO score ≥0.4, and (3) Hardy-Weinberg equilibrium *P*≥1×10^−5^. We used the minor allele dosage effect (homozygous for derived allele=dosage score 2) to study the interaction between daily sodium consumption and glycosaminoglycan genes.

### Statistical Analysis

Continuous data were expressed as mean and SD for parametric data. The median and interquartile range, visualized with values for quartile 1 (Q1) and quartile 3 (Q3), were used for nonparametric variables. For MAP, 2 linear regression models were corrected for age, age^2^, sex, and BMI. The first model was fitted with estimated 24-hour sodium intake and SNP (minor allele dosage score) as independent variables, and the second model was the same as the first model but also included an interaction term for estimated 24-hour sodium intake and SNP. The likelihood ratio test was used to test the significance of adding the interaction term to the model. A false discovery rate adjusted *P* value of ≤0.05 of this likelihood ratio test was considered significant and indicated an interaction between the SNP and estimated 24-hour sodium intake. SNPs that showed a significant interaction with both formulas for estimated sodium intake on BP in the discovery cohort were also selected in UKBB to explore the interaction. The above analyses were also replicated for systolic and diastolic BP. Furthermore, a sensitivity analysis was performed for MAP with only individuals without antihypertensive medication. Statistical analyses were performed using RStudio (R version 4.1.3; RStudio Team). METAL analysis software^32^ was used to perform a meta-analysis. *P* values and direction of effect, weighted according to sample size, were used to combine *P* values without genomic control correction.

### Multiethnic Replication Analysis

The top SNPs in the meta-analysis were validated using baseline data of the HELIUS cohort to assess coherence across different genetic ancestries. The HELIUS study is an ongoing prospective cohort study with baseline visits between 2011 and 2015 in Amsterdam, the Netherlands. The study design and aims of this study have previously been described elsewhere.^33^ Briefly, based on the municipality register of Amsterdam, people aged 18 to 70 years were randomly invited to participate, stratified by ethnic origin (Dutch, Surinamese, Ghanaian, Turkish, or Moroccan). Recruitment and the definition of ethnic origin are described in Appendix S2. This study measured BP after 5 minutes of seated rest using a validated semiautomatic oscillometric device (Microlife WatchBP Homel; Microlife AG) and appropriate cuff sizes. For the validation analysis, we included participants with available data for BP and estimated sodium intake using ethnicity-specific FFQs (Food Frequency Questionnaires), which were administered to a group of 5084 participants (not in Ghanaian origin participants).^34^ We excluded those with implausible calorie intake, those who filled in the wrong ethnicity-specific FFQ based on ethnicity, and those with very high or very low sodium intake (Appendix S3).^35^ We further selected participants for whom genetic data were available. In total, 2239 participants could be included in the analyses (522 of South-Asian Surinamese, 296 of African Surinamese, 452 of Turkish, 607 of Moroccan, and 362 of Dutch origin). By using the Dutch Food Composition Table, sodium intake was estimated from these questionnaires as is described in Appendix S3.^36^ The medical ethical review board of the Amsterdam University Medical Center, location Academic Medical Center, approved the HELIUS study. All participants provided written informed consent before data collection, and the study was conducted according to the principles of the Declaration of Helsinki. The HELIUS board approved the current analysis (210925).

### Functional Relevance of Identified Glycosaminoglycan Genes for Glycosaminoglycan Composition and Salt-Sensitive BP Response

To investigate whether the identified genotypes affect the synthesis of glycosaminoglycan disaccharides, we selected Dutch origin HELIUS participants to analyze urinary glycosaminoglycan composition (ie, urinary excretion of heparan sulfate disaccharides (D0A0 [ΔUA-GlcNAc], D0S0 [ΔUA-GlcNS], D0A6 [ΔUA-GlcNAc6S], D2A0 [ΔUA2S-GlcNAc], D0S6 [ΔUA-GlcNS6S], D2S0 [ΔUA2S-GlcNS]), dermatan sulfate disaccharides (D0a10 [ΔUA-GalNAc4S6S] and D0a4 [ΔUA-GalNAc4S]). In spot urine samples, we measured urinary glycosaminoglycan excretion with a validated high-performance liquid chromatography with mass spectrometry/mass spectrometry and adjusted the disaccharide concentrations for creatinine concentrations of the urine samples.^37,38^ Within the Dutch participants, we excluded all active smokers, participants with diabetes (based on self-report or increased fasting glucose [≥7 mmol/L] or use of glucose-lowering medication) or chronic kidney disease (The Kidney Disease: Improving Global Outcomes [KDIGO] stage III t/m IV or microalbuminuria ≥20 mg/L), and participants using antihypertensive medication or steroids (anatomical therapeutic chemical codes C02, CO3, C07–C09, H02, and D07). We stratified for rs2892799 (CC, CT, and TT). With 1:1 nearest neighbor matching without replacement, we matched 19 individuals from each group based on sex, age, BMI, systolic BP (SBP), diastolic BP, smoking, and kidney function (estimated glomerular filtration rate, Chronic Kidney Disease Epidemiology Collaboration [CPD-EPI]). This resulted in 57 individuals in which urinary glycosaminoglycan composition could be analyzed.

To assess differences in glycosaminoglycan composition between individuals with a salt-sensitive BP rise after a high-sodium diet and salt-resistant subjects, we measured 24-hour urinary glycosaminoglycan excretion in 24-hour urine after a dietary sodium load of 7 days. Also in these samples, the glycosaminoglycan concentration was adjusted for the creatinine concentrations. This study was conducted in the Amsterdam University Medical Center, location Academic Medical Center, the Netherlands, after approval of the local ethics committee (Dutch Trial register ID NL3933). Twelve nonsmoking men between 18 and 40 years old, with BP below 140/90 mm Hg, BMI <30 kg/m^2^, and normal renal function (defined as creatinine clearance >60 mL/min and absence of proteinuria) were subjected to a 7-day low-sodium diet (<50 mmol sodium per day)—which was considered as the baseline—and a 7-day high-sodium diet (>200 mmol sodium per day), in randomized order. Between the diets, there was a washout period of 7 to 14 days before participants switched to the other diet. After each diet, brachial BP was measured in a supine position after at least 10 minutes of supine rest. With a validated semiautomatic device (Omron 705-IT; OMRON Healthcare), we performed 5 sequential measurements, of which the mean of the last 2 readings was used to identify a salt-sensitive BP response (defined in our study as ≥1 mm Hg MAP increase by comparing BP after the low and high-sodium diet). Written informed consent was obtained from all subjects, and the study was conducted in accordance with the Declaration of Helsinki.

### Data Availability Statement

The data supporting this study’s findings are available from EPIC-Norfolk, UK Biobank, and HELIUS. Restrictions apply to the availability of these data, which were used under license for this study. Researchers interested in access to the data may contact these individual organizations. Raw data from the sodium intervention trial can be obtained via the corresponding author (L. Vogt) upon reasonable request.

### Code Availability Statement

All code for data cleaning and analysis associated with this publication can be obtained via the corresponding author (L. Vogt) upon reasonable request.

## RESULTS

### Characteristics of Study Participants

The selection of participants from EPIC-Norfolk and UKBB is shown in Figure S1. A total of 20 420 participants from EPIC-Norfolk and 414 132 from UKBB were included. The baseline characteristics of the participants in both cohorts are presented in Table 1.

**Table 1.**
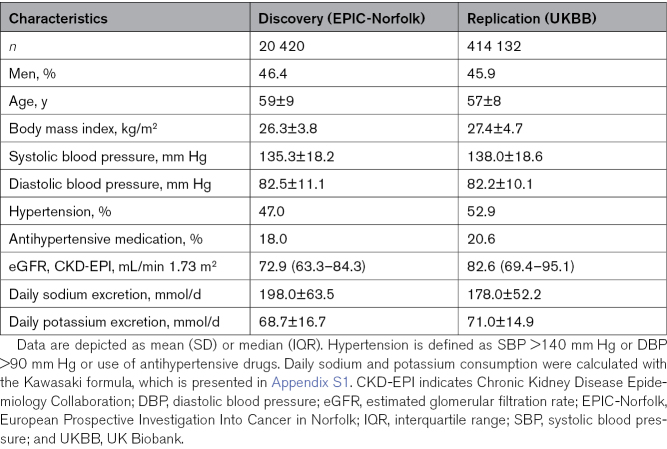
Characteristics of Study Populations

The percentage of men in EPIC-Norfolk and UKBB (46.6% versus 45.9%) and the mean age (59 years versus 57 years) were comparable. BMI was higher in UKBB (26.3 [SD 3.8] versus 27.4 [SD 4.7] kg/m^2^), as was the percentage of participants using antihypertensive medication (18.0% versus 20.6%). In UKBB, participants had lower estimated 24-hour sodium excretion (198.0 [SD 63.5] versus 178.0 [SD 52.2] mmol/day) and higher estimated 24-hour potassium excretion (68.7 [SD 16.7] versus 71.0 [SD 14.9] mmol/day).

### Selected SNPs for Analysis

Reactome (date of access: June 28, 2022) revealed 124 proteins involved in glycosaminoglycan metabolism. Literature research revealed 6 (*EXTL1*, *EXTL2*, *EXTL3*, *FAM20B*, *SULF1*, and *SULF2*) additional relevant genes, which were added to our analysis.^39–41^ Table S1 depicts all selected genes and their corresponding genomic locations used in our study. The genetic locations (±10 kbp) contained 200 990 unique SNPs in the EPIC-Norfolk cohort, of which 54 126 SNPs met our thresholds for quality control (Figure S2).

### Sodium and Glycosaminoglycan SNP Interaction on BP

**Figure 1.**
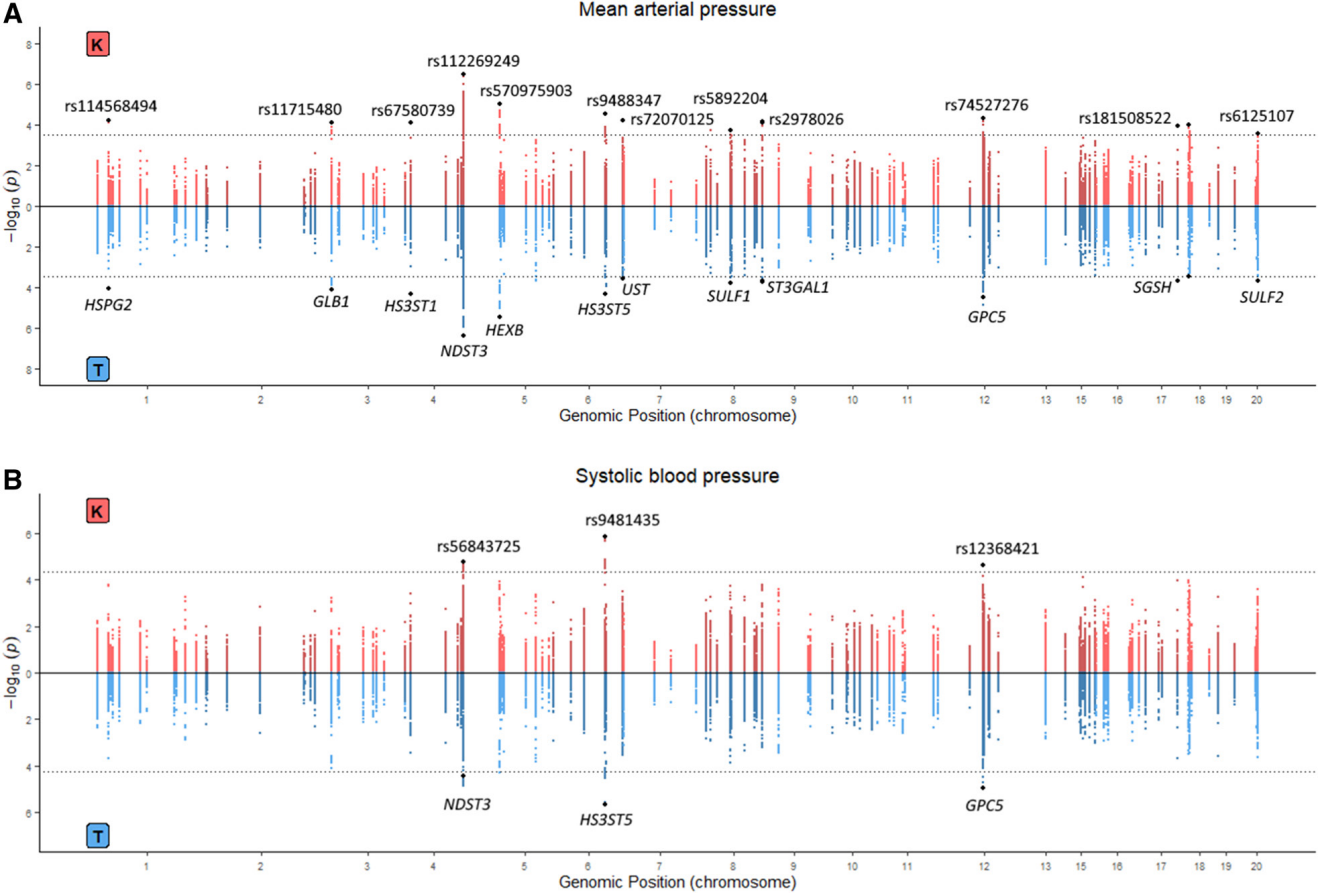
**Miami plots showing variants which have a significant interaction with sodium on blood pressure. A**, Miami plot for mean arterial pressure results of EPIC-Norfolk (European Prospective Investigation Into Cancer in Norfolk). **B**, Miami plot for systolic blood pressure results of EPIC-Norfolk. The significance of the interaction term of individual single-nucleotide polymorphisms over the model without the interaction term is visualized. The upper part of the graphs shows the significance for sodium intake estimated by the Kawasaki formula. The lower part of the graphs shows the results with estimated sodium intake using the Tanaka formula. Single-nucleotide polymorphism rsIDs as well as the corresponding glycosaminoglycan gene genomic region are visualized. The dotted line represents the false discovery rate <0.05 significance level. K indicates Kawasaki; rsID, reference single-nucleotide polymorphism identification number; and T, Tanaka.

In the discovery cohort, 282 SNPs showed a significant interaction with both Kawasaki- and Tanaka-based estimated 24-hour sodium intake on MAP (Figure 1A). For diastolic BP and SBP, 323 and 53 significant variants were found, respectively (Figure S3; Figure 1B). The top SNPs of each glycosaminoglycan gene are depicted in Table S2. A flowchart showing the significance of SNPs in the discovery (Kawasaki) and interval validation (Tanaka) phase and the total number of SNPs analyzed in UKBB is visualized in Figure S4.

Of the significant SNPs for MAP, 269 variants were also available in UKBB. The meta-analysis of these variants in both cohorts showed a false discovery rate significant *P* value for 111 SNPs on chromosome 4 (Table S3). Conditional analysis revealed that these 111 SNPs were represented by 1 signal in which rs2892799 represented the strongest association (Table 2). None of the significant SNPs for diastolic BP (317 variants available) remained significant in the meta-analysis combining EPIC-Norfolk and UKBB. For SBP, 51 variants were also available in UKBB, all of which remained significant in the meta-analysis (Table S4). These variants were represented by 3 signals: rs56843725, rs4438821, and rs9654628 (Table 2). Of these signals, rs56843725 and rs4438821 were also represented by the signal of rs2892799 for MAP (Table S3). In the sensitivity analysis, which included only participants without antihypertensive medication, no significant SNPs could be observed for MAP.

**Table 2.**
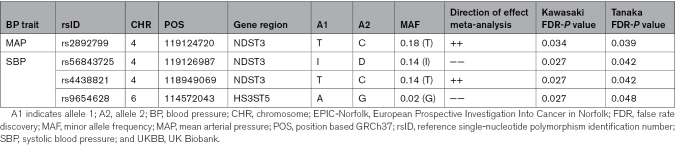
Results of the Meta-Analysis of EPIC-Norfolk and UKBB

### Multiethnic Replication of Significant Variants With FFQ Estimated Sodium Intake Tertiles

For our multiethnic replication in the HELIUS study, 2239 genotyped participants with complete BP and sodium intake data were available (Figure S5). Of these participants, the majority were of Moroccan (27.1%) and South-Asian Surinamese (23.3%) descent (Table 3; Table S5 for characteristics per ethnic group). The mean age was 47 (SD 12) years, which is lower than the mean age in EPIC-Norfolk and UKBB. The mean BMI was 27.2 (SD 4.9) kg/m^2^, comparable to the other cohorts. Of the participants, 18.1% used antihypertensive medication.

**Table 3.**
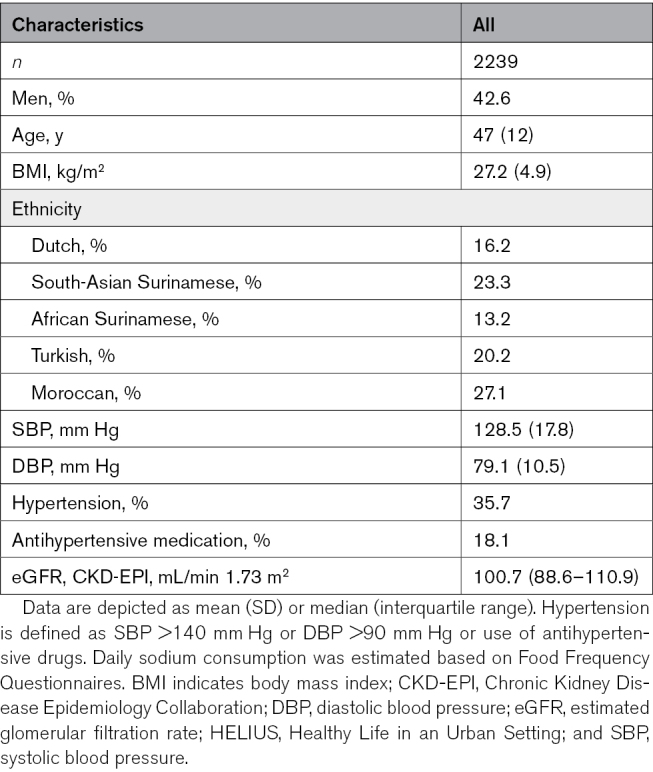
Characteristics of Multiethnic HELIUS Cohort

**Figure 2.**
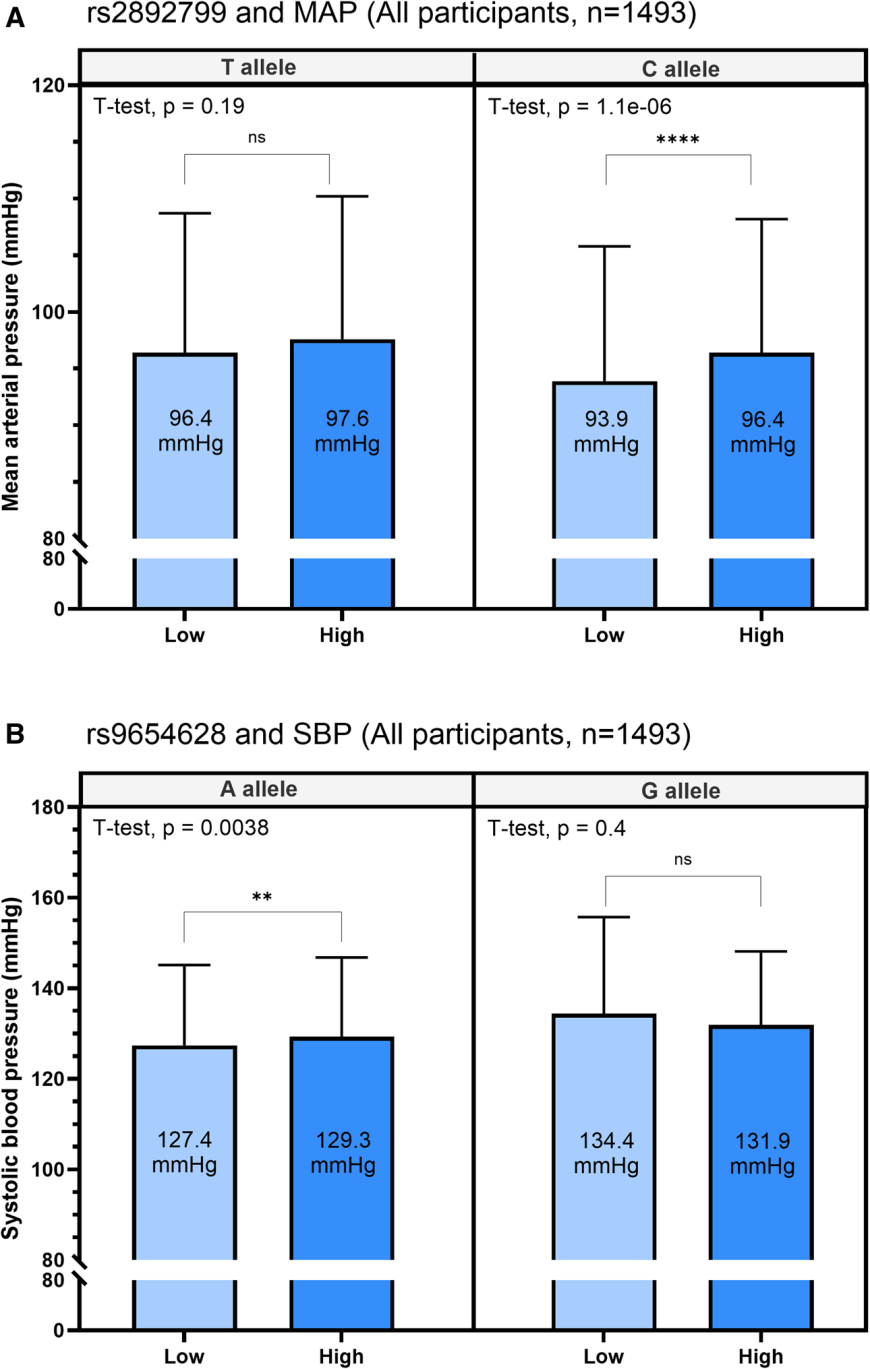
**Blood pressure stratified for rs2892799 and rs9654628 alleles and sodium intake estimated with Food Frequency Questionnaires in the multi-ethnic HELIUS (Healthy Life in an Urban Setting) cohort. A**, Mean arterial pressure stratified for rs2892799 alleles and estimated sodium intake. **B**, Systolic blood pressure stratified for rs9654628 alleles and sodium intake. Error bars represent the SD. For rs2892799: within the high and low sodium tertile, 123 subjects were homozygous TT, 518 heterozygotes, and 852 homozygous CC. For rs9654628: within the high and low sodium tertile, 1340 subjects were homozygous AA, 148 were heterozygotes, and 5 were homozygous GG. MAP indicates mean arterial pressure; and SBP, systolic blood pressure.

We stratified the subjects according to the alleles of significant SNPs: rs2892799 (T and C) and rs9654628 (A and G). rs56843725 and rs4438821 were not genotyped in the HELIUS study. We further stratified this multiethnic cohort according to the highest and lowest tertiles of sodium consumption (low and high subgroups). We observed significantly higher SBP for the A allele of rs9654628 during high sodium intake but not for the G allele (Figure 2A; Figure S6 stratified for ethnicity). For rs2892799, we observed higher MAP for the C allele after high sodium intake, while no effect of sodium was observed for the T allele (Figure 2B; Figure S7 stratified for ethnicity).

### Relevance of the N-Sulfated D0S0 Heparan Sulfate Epitope in the Salt-Sensitive BP Response

**Figure 3.**
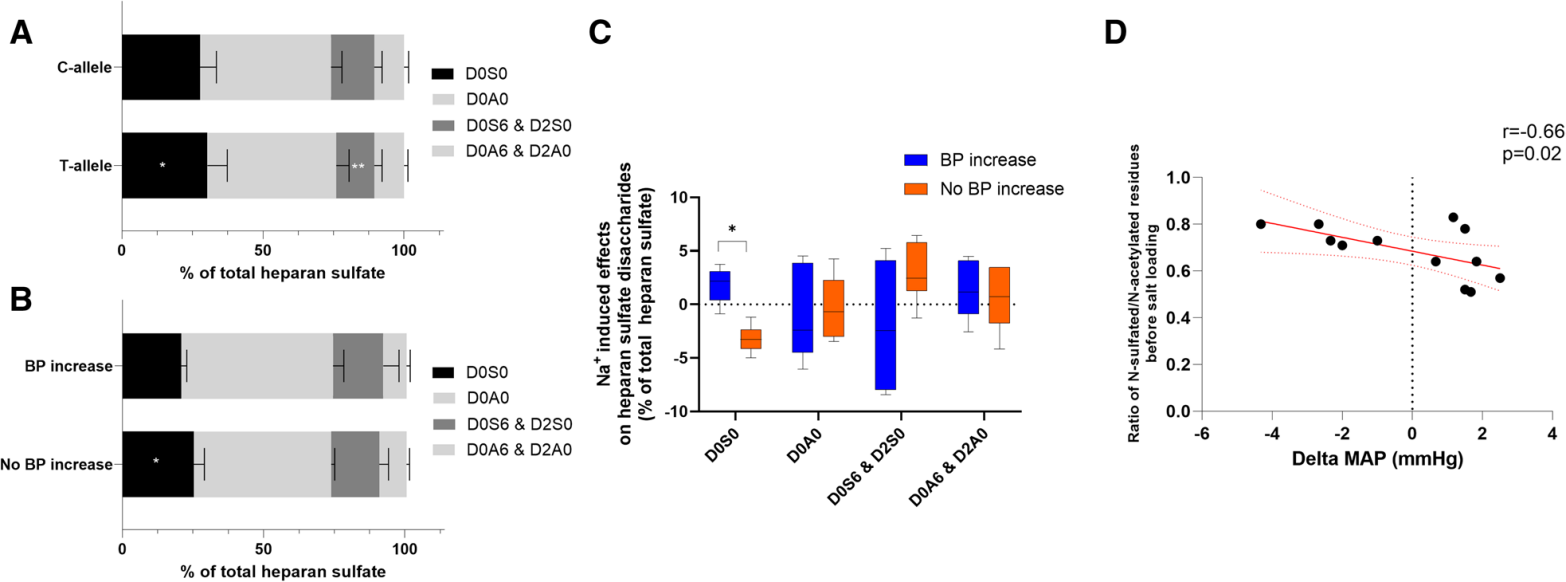
**Heparan sulfate expression in relation to rs2892799 genotype and blood pressure (BP) phenotypes. A**, Expression of heparan sulfate disaccharides in spot urine of HELIUS (Healthy Life in an Urban Setting) participants stratified for rs2892799 genotype. The *X* axis represents the median percentage of the individual disaccharides with regard to total heparan sulfate. The error bar represents the interquartile range. **B**, Expression of heparan sulfate disaccharides in 24-hour urine after a 7-day low (50 mmol Na^+^ per day) and 7-day high (200 mmol Na^+^ per day) diet stratified for individuals with BP increase and individuals with stable BP. The *x* axis represents the median percentage of the individual disaccharides with regard to total heparan sulfate. The error bar represents the interquartile range. **C**, Sodium-induced effects on the median percentage of the individual disaccharides with regard to total heparan sulfate stratified for individuals with BP increase after sodium loading and individuals with no BP increase. **D**, A significant correlation exists between the ratio of N-sulfated/N-acetylated residues before sodium loading and BP response to sodium (r=−0.66; *P*=0.02). The dotted red line represents the error of confidence of the regression. The notation of glycosaminoglycan disaccharides is as follows^38^: D0A0=ΔUA-GlcNAc, D0S0=ΔUA-GlcNS, D0A6=ΔUA-GlcNAc6S, D2A0=ΔUA2S-GlcNAc, D0S6=ΔUA-GlcNS6S, D2S0=ΔUA2S-GlcNS. *P<0.05 when compared with individuals with BP increase. MAP indicates mean arterial pressure.

rs2892799 is an intron variant that is mapped within the *NDST3* (heparan sulfate N-deacetylase/N-sulfotransferase 3) gene. *NDST3* belongs to the family of *NDST* genes (*NDST 1-4*), coding for bifunctional enzymes that catalyze N-deacetylation and N-sulfation of *N*-acetylglucosamine residues in heparan sulfate. Of the 2239 participants used in the analyses, we selected 57 Dutch-originated HELIUS participants based on the rs2892799 genotype (Figure S8). After stratification by rs2892799 alleles, we observed different expressions of N-sulfated heparan sulfate epitopes between C and T alleles. The T allele showed higher expression of D0S0 as a proportion of heparan sulfate and lower expression of D0S6&D2S0 than the C allele (Figure 3A).

Next, we performed a randomized sodium intervention trial in healthy male volunteers to investigate differences in sodium-induced effects on heparan sulfation patterns. In this trial, we measured N-sulfated (D0S0 and D0S6&D2S0) and N-acetylated (D0A0 and D0A6&D2A0) heparan sulfate-glucosamine residues in 24-hour urine (Figure 3B through 3D) of 12 normotensive men with a mean age of 23 (4) years, normal kidney function (creatinine 79.0 [12.1] μmol/L), and body weight (75.7 [6.8] kg). After successful dietary sodium loading, BP increased in 6 participants (Table S6). After the low-sodium diet, we observed a lower proportion of N-sulfated D0S0 in subjects showing a salt-sensitive BP increase (Figure 3B). After sodium loading, we observed differences between salt-resistant and salt-sensitive individuals in the expression of N-sulfated D0S0, while no differences in N-acetylated residues could be observed (Figure 3C). Also, the ratio of N-sulfated/N-acetylated residues before sodium loading shows a significant negative correlation with high sodium-induced changes in MAP independent of a definition for salt sensitivity (Figure 3D). rs9654628 is an intron variant mapped to *HS3ST5* (heparan sulfate-glucosamine 3-sulfotransferase 5), an enzyme involved in 3-*O*-sulfation of glucosamine residues.^42^ 3-*O*-sulfation could not be measured in this study using high-performance liquid chromatography with mass spectrometry/mass spectrometry.

## DISCUSSION

In this candidate gene study, we explored interactions between 54 126 variants in 130 glycosaminoglycan genes and sodium on BP in >436 000 individuals. We show that in individuals carrying the C allele of rs2892799, the difference in MAP between those in high and low sodium conditions is more extensive when compared with individuals carrying the reference allele (T). In a multiethnic validation cohort, we showed that the C allele was associated with a 2.7% higher MAP in individuals with high sodium intake than in individuals with low sodium intake. In comparison, this was 1.2% for the T allele. Variant rs2892799 is mapped to an intronic region in the *NDST3* gene, a bifunctional enzyme involved in both N-deacetylation and N-sulfation of *N*-acetylglucosamine residues in heparan sulfate and heparin.^43^ We further showed in the observational HELIUS cohort that the N-sulfated D0S0 heparan sulfate epitope expression was 9.1% higher for the T-allele genotype. Conversely, our time-controlled sodium intervention study of D0S0 expression in response to high sodium intake showed an increased expression of D0S0 in salt-sensitive individuals and a decrease in salt-resistant individuals. In our multiethnic cohort, we also validated the association between SBP and a variant mapped to *H3ST5* (rs9654628), resulting in a 1.5% higher SBP during high sodium intake for the A allele, while for the G allele, the difference was −1.9%. These results might imply an important role for glycosaminoglycan metabolism genes—*NDST3* and *H3ST5* especially—in sodium-related BP regulation.

NDST enzymes are vital in determining sulfation motifs of the developing heparan sulfate chain.^44,45^ NDST3 shows high deacetylase activity and low sulfotransferase activity.^46^ The Genotype-Tissue Expression (GTEx) database shows rs2892799 is associated with increased NDST3 expression in thyroid tissue of homozygotes of the derived C allele (n=574; *P*=3.0×10^−8^).^47^ This might, regarding the biological activity of NDST3, result in reduced sulfation of heparan sulfate for C allele associated genotypes. With regard to sodium homeostasis, the amount of sulfate groups is highly important because sulfate groups strongly increase the negative charge density of glycosaminoglycans and thereby determine interactions with positively charged ions such as sodium.^20,48^ In patients with heart failure—known to have high skin sodium content—it was shown that skin biopsies contained a 56% higher amount of sulfated glycosaminoglycans and increased sulfation per disaccharide compared with healthy controls.^49,50^ This is in line with the observation that aquatic invertebrates increase the amount of sulfated glycosaminoglycans as a function of their habitat’s salinity.^12^ The exact role of changed sulfation of thyroid heparan sulfate by rs2892799 on thyroid function is unclear. However, thyroid function is associated with the expression of heparan sulfate in tissues.^51^ In skin and muscle, expression of both NDST3 mRNA and protein is low.^52,53^ The GTEx database also shows increased serine protease 12 (PRSS12) expression in skin tissue of rs2892799 C-allele homozygotes (n=517; *P*=3.9×10^−7^).^47^ *PRSS12* encodes the serine protease neurotrypsin, representing an *NDST3* neighboring gene on chromosome 4. Agrin, a major heparan sulfate proteoglycan, can be cleaved by neurotrypsin at 2 homologous, highly conserved sites.^54^ The function of the cleavage of agrin by neurotrypsin has been mainly investigated in the central nervous system. It has been shown to be important in synapse formation, maintenance and plasticity, and the maturation of the neuromuscular junction.^55,56^ Neurotrypsin and agrin have not previously been associated with BP or salt sensitivity. Agrin was included in this analysis but did not show a significant interaction with sodium on BP.

Although follow-up studies are needed to investigate the exact working mechanism of rs2892799, we observed lower expression of D0S0 for the C allele and salt sensitives from our sodium intervention study. Concomitantly, the C allele was associated with salt sensitivity in our candidate gene study. These data suggest that variant rs2892799 might influence the N-sulfation of heparan sulfate and thereby affect the interaction with sodium on BP. Because we measured urinary glycosaminoglycan expression, follow-up studies are needed to investigate the effect of rs2892799 on sulfation patterns of heparan sulfate in different tissues and how this effect is mediated.

Previous GWASs have revealed several loci showing interaction with sodium on BP. However, experimental evidence and biological relevance of these loci in BP regulation are often lacking.^57–60^ These studies investigated over 17 500 individuals, yet all were of Asian descent, and results were not validated in other ethnic backgrounds. Li et al^60^ showed an interaction of a UST (uronyl 2-sulfotransferase) variant with sodium intake on BP in an Asian population. Like NDST3 and HS3ST5, UST is a sulfotransferase enzyme that transfers sulfate to the 2-position of uronyl residues of dermatan and chondroitin sulfate. No associations with *UST* variants could be found in our study in which other ethnicities were included. This might imply that the interaction between glycosaminoglycans and sodium differs between ethnic groups. According to the allele frequencies observed in the 1000 Genomes Project, the derived C allele of rs2892799 is the major allele in European, American, and Asian populations. In contrast, the T allele is the major allele in African populations.^61^ Because our data suggest the C allele is associated with salt sensitivity, this might contrast with the high prevalence of salt sensitivity in people of African descent. Therefore, studies investigating tissue sodium and genotype interaction should be performed in different ethnic groups.

Previous non-BP-focused GWASs have shown associations between *NDST3* and the development of mental disorders such as schizophrenia, anxiety, and bipolar disorders.^62–64^ No previous BP or salt sensitivity associations were reported. Other NDST isoforms have been investigated in animal studies to explore the effect of N-sulfation on vascular health and endothelial function.^65,66^ An in vitro study showed that proinflammatory cytokines can increase NDST expression, leading to increased sulfation of heparan sulfate.^67^ In humans, it was also demonstrated that monocyte influx and concomitant alterations in lymphatic skin vasculature are pivotal for adequate BP regulation in high sodium conditions.^68^ These data suggest that NDST activity and N-sulfation of heparan sulfate might not only affect sodium homeostasis by increasing the amount of negatively charged sulfate groups in tissues (and thereby increasing salinity tolerance) but possibly also by modulating monocyte activation/migration and affecting the immunologic pathways involved in salt sensitivity.

For SBP, we observed an interaction for 2 *NDST3* variants, of which rs2892799 showed the strongest association in the conditional analysis. Besides, an interaction between 24-hour sodium intake and rs9654628 (mapped to *HS3ST5*) on SBP was observed. HS3ST5 belongs to a group of heparan 3-*O*-sulfotransferases and is involved in generating anticoagulant active heparan sulfate (heparin).^69^ Oral heparin treatment prevented the increase in SBP in rats on a high-sodium diet.^70^ Noticeably, like NDST isotypes, heparan 3-*O*-sulfotransferase isotypes might also affect BP via their crucial role in endothelial glycocalyx structure and subsequent vascular homeostasis.^71^ Indeed, glycocalyx restoration with glycosaminoglycan supplementation has shown to reduce BP in multiple controlled clinical trials—an effect that may directly be explained by the interaction between sodium and the endothelium.^18,72,73^

To our knowledge, this is the first candidate gene approach studying the role of glycosaminoglycan metabolism in the salt sensitivity of BP. We showed an interaction between 2 variants (rs2892799 and rs9654628) on BP, and we replicated these findings in 3 cohorts, of which 1 was a multiethnic cohort, with a total of over 436 000 individuals. Furthermore, as we acknowledge difficulties in estimating sodium intake with random casual urine samples, we used 2 different formulas together with FFQ to estimate 24-hour sodium intake. We only selected variants for replication, which showed significance for both formulas. Also, we could show the biological relevance and importance of our variant in heparan sulfate composition and salt sensitivity by a dietary sodium intervention. However, certain limitations of our analysis need to be considered. With this candidate gene approach, we selectively investigated over 200 000 variants of our interest. Therefore, other mechanisms in the relationship between sodium and BP cannot be discovered. However, with this design, in contrast to, for example, GWASs, we observed small BP effects after correction for other confounders such as age, sex, and BMI. Also, the complexity of glycosaminoglycan metabolism might suggest a more complex polygenetic interaction between sodium and glycosaminoglycan genes on BP, which is not investigated in this study. Different variants might also be discovered when a large multiethnic cohort is used as a discovery cohort. Finally, the relationship between glycosaminoglycan-mediated sodium accumulation and BP extends beyond glycosaminoglycans and involves immune system pathways and new lymph vessel formation.^68^ Genetic variants in these pathways might also influence the salt sensitivity of BP.

## Perspectives

This study shows for the first time that genetic variation in metabolic glycosaminoglycan adaptation enzymes is linked to the salt sensitivity of BP in humans. Variants in 2 genes (*NDST3* and *HS3ST5*) show an interaction with sodium consumption on BP in 2 European and 1 multiethnic cohort. Furthermore, we were able to show that genotypes of the *NDST3* variant (rs2892799) show different expression of D0S0 heparan sulfate epitope in urine. This epitope shows, irrespective of genotype, a different expression between salt-sensitive and salt-resistant individuals after dietary sodium loading. In line with earlier observations from controlled clinical trials, our study presents new evidence that targeting glycosaminoglycan metabolic adaptation may prove to negate the adverse long-term effects of high-salt consumption. Further studies are needed to investigate how the interaction between sodium consumption and glycosaminoglycans relates to other recently uncovered crucial pathways contributing to hypertension development, such as proinflammatory, gut microbiomal, and metabolomic alterations.

## ARTICLE INFORMATION

### Acknowledgments

This research has been conducted using the EPIC-Norfolk (European Prospective Investigation Into Cancer in Norfolk) cohort under application number ENDR028-2020. The authors are grateful to all the participants who have been part of the project and the many members of the study teams at the University of Cambridge who have enabled this research. The UK Biobank Resource was used under application number 75038. The authors are grateful to all the participants and dedicated staff members based in multiple locations across the United Kingdom. Data from the HELIUS study (Healthy Life in an Urban Setting) were used under application numbers 210925 and 200605. The authors are grateful to all the participants and dedicated staff members who have enabled this research. The authors thank Dr Mary Nicolaou for collecting data on dietary patterns with Food Frequency Questionnaires and helping us analyze sodium intake in HELIUS participants.

### Author Contributions

H. Galenkamp, A.H. Zwinderman, and B.-J.H. van den Born are involved in the design and data collection of the HELIUS study. J.J. Oppelaar, M. A. Romman, and G. N. Sahebdin analyzed sodium intake using the FFQs (Food Frequency Questionnaires) of the HELIUS study. R.H.G.O. Engberink and J.J. Oppelaar conducted the sodium intervention trials in volunteers and analyzed the data. J.J. Oppelaar and B. Ferwerda performed the statistical analyses in EPIC-Norfolk, UK Biobank, and HELIUS. S.M. Boekholdt supervised the analysis in EPIC-Norfolk. J.J. Oppelaar and L. Vogt interpreted the data and drafted the manuscript. All authors reviewed the manuscript and approved the final version. L. Vogt supervised the sodium intervention trials and the analysis performed in this study.

### Sources of Funding

This work was supported by funding from the Dutch Kidney Foundation (Senior Kolff grant number 18OKG12 to L. Vogt).

The EPIC-Norfolk study (European Prospective Investigation Into Cancer in Norfolk) has received funding from the Medical Research Council (MR/N003284/1 and MC-UU_12015/1) and Cancer Research UK (C864/A14136). The genetics work in the EPIC-Norfolk study was funded by the Medical Research Council (MC_PC_13048).

The UK Biobank has received funding from the Wellcome Trust and UK Medical Research Council, the Department of Health, the Scottish Government, the Northwest Regional Development Agency, the British Heart Foundation, and Cancer Research UK.

The Academic Medical Center of Amsterdam and the Public Health Service of Amsterdam (GGD Amsterdam) provided core financial support for HELIUS (Healthy Life in an Urban Setting). The HELIUS study is also funded by research grants from the Dutch Heart Foundation (Hartstichting; grant No. 2010T084), the Netherlands Organization for Health Research and Development (ZonMw; grant No. 200500003), the European Integration Fund (grant No. 2013EIF013), and the European Union (Seventh Framework Program grant No. 278901).

### Disclosures

None.

## Supplementary Material


