# Supplementary material for: Genetic Variance in Heparan Sulfation Is Associated With Salt Sensitivity
Source: Hypertension. 2024 Sep 9;81(10):2101–12. doi: 10.1161/HYPERTENSIONAHA.124.23421 (PMC11404764; doi:10.1161/HYPERTENSIONAHA.124.23421)
Supplement: Supplementary file 1 [file hyp-81-2101-s001.doc]

**supplemental material**

**Title:** Genetic variance in heparan sulfation is associated with salt-sensitivity

**Authors:** Jetta J. Oppelaar^1,2^, Bart Ferwerda^3^, Mohamed A. Romman^1^, Ghazalah N. Sahebdin^1^, Aeilko H. Zwinderman^3^, Henrike Galenkamp^4^, S. Matthijs Boekholdt^5,6^, Bert-Jan H. van den Born^4,6,7^, Rik. H.G. Olde Engberink^1,2^, Liffert Vogt^1,2^

**Affiliations:**

^1^ Amsterdam UMC location University of Amsterdam, Department of Internal Medicine, Section of Nephrology, Meibergdreef 9, Amsterdam, The Netherlands

^2^ Amsterdam Cardiovascular Sciences, Microcirculation, Amsterdam, The Netherlands

^3^ Amsterdam UMC location University of Amsterdam, Department of Clinical Epidemiology and Biostatistics, Meibergdreef 9, Amsterdam, The Netherlands

^4^ Amsterdam UMC location University of Amsterdam, Department of Public and Occupational Health, Amsterdam Public Health (APH), Meibergdreef 9, Amsterdam, The Netherlands

^5^ Amsterdam UMC location University of Amsterdam, Department of Cardiology, Meibergdreef 9, Amsterdam, The Netherlands

^6^ Amsterdam Cardiovascular Sciences, Atherosclerosis & ischemic syndromes, Amsterdam, The Netherlands

^7^ Amsterdam UMC location University of Amsterdam, Department of Internal Medicine, Section of Vascular Medicine, Meibergdreef 9, Amsterdam, The Netherlands

**Address for Correspondence:** Liffert Vogt MD PhD, Amsterdam UMC, Department of Internal Medicine, Section of Nephrology, Room D3-324, P.O. Box 22660, Meibergdreef 9, 1100 DD Amsterdam, The Netherlands, E-mail: [l.vogt@amsterdamumc.nl](mailto:l.vogt@amsterdamumc.nl)

**Supplemental Text:**

**Appendix 1.** Kawasaki and Tanaka formula to estimate 24-h sodium excretion

**Appendix 2.** Selection of study participants of the HELIUS study

**Appendix 3.** Estimation of sodium intake in the HELIUS study

**Supplemental Tables:**

**Table S1.** Glycosaminoglycan genes included in our analysis with corresponding chromosomal position (GrCh 37)

**Table S2.**  Significant top SNPs for both formulas in EPIC Norfolk and their corresponding gene regions

**Table S3.**  Significant SNPs for MAP from meta-analysis combining EPIC Norfolk and UK biobank

**Table S4.**  Significant SNPs for SBP from meta-analysis combining EPIC Norfolk and UK biobank

**Table S5.**  Baseline characteristics of the participants of the HELIUS cohort stratified for ethnicity

**Table S6.** Characteristics of study participants stratified for BP response after HSD

**Supplemental Figures:**

**Figure S1.** Flowchart of inclusion

**Figure S2**. Flowchart of selected SNPs in EPIC Norfolk based on quality control

**Figure S3**. Miami plots for diastolic blood pressure results of EPIC Norfolk.

**Figure S4.** Flowchart of SNPs selected for replication in UK biobank

**Figure S5.** Flowchart for selection of participants in the HELIUS study. FFQ, food frequency questionnaire

**Figure S6.** Systolic BP stratified for alleles of rs9654628 and sodium intake estimated with food frequency questionnaires for every ethnic group in the HELIUS population stratified for ethnicity

**Figure S7.** Mean arterial pressure for alleles of rs2892799 and sodium intake estimated with food frequency questionnaires for every ethnic group in the HELIUS population stratified for ethnicity.

**Figure S8.** Flowchart for selection Dutch HELIUS participants based on genotype. FFQ, food frequency questionnaire. KDIGO, Kidney Disease: Improving Global Outcomes

**Supplemental Text**

**Appendix 1**

**Kawasaki formula:**

Estimated 24-hour urine sodium excretion (mmol/day) =

16.3 × ((spot urine sodium (mmol/L) / (spot urine creatinine (mg/dL) × 10)

× estimated 24‐hour urinary creatinine (mg/day))^0.5

Estimated 24-hour urine potassium excretion (mmol/day) =

7.2 × ((spot urine potassium (mmol/L) / (spot urine creatinine (mg/dl) × 10)

× estimated 24‐hour urinary creatinine (mg/day))^0.5

Estimated 24-hour urinary creatinine (mg/day)

Men =

−12.63×age (years)+15.12 ×weight (kg)+7.39 ×height (cm)−79.9

Women =

−4.72×age (years)+8.58 ×weight (kg)+5.09 ×height (cm)−74.5

**Tanaka formula:**

Estimated 24-hour urine sodium excretion (mmol/day) =

21.98 × ((spot urine sodium (mmol/L) / (spot urine creatinine (mg/dL) × 10)

× estimated 24‐hour urinary creatinine (mg/day))^0.392

Estimated 24-hour urine potassium excretion (mmol/day) =

7.59 × ((spot urine sodium (mmol/L) / (spot urine creatinine (mg/dL) × 10)

× estimated 24‐hour urinary creatinine (mg/day))^0.431

Estimated 24-hour urinary creatinine (mg/day) =

−2.04 × age (years) + 14.89 × weight (kg) + 16.14 × height (cm) − 2244.45

**Appendix 2**

Through the municipality register of Amsterdam, citizens in the range of 18-70 years were randomly sampled, stratified by ethnic origin. The municipality register of Amsterdam contains data on country of birth of citizens and of their parents, enabling sampling based on the widely accepted Dutch standard indicator for ethnic origin. The country of birth indicator was chosen for its objectivity and stability over time. Cross-validation studies demonstrated a strong correlation between the country of birth indicator and self-identified ethnic group indicators, particularly among Turkish, Moroccan and Surinamese individuals in the Netherlands.

For the purpose of identifying individuals of non-Dutch ethnic origin, two criteria were applied:

(1) Those born outside the Netherlands with at least one parent born outside the Netherlands (first generation) *or*

(2) Those born in the Netherlands with both parents born outside the Netherlands (second generation)

In case of the Dutch sample, participants were limited to individuals born in the Netherlands with parents also born in the Netherlands.

A limitation of the country of birth indicator for ethnicity is that people who are born in the same country might have a different ethnic background. This is particularly relevant in the context of the Surinamese population. Consequently, after data collection, participants of Surinamese ethnic origin underwent further classification based on self-reported ethnic origin obtained through a questionnaire. The categories included ‘African’, ‘ South-Asian’, ‘ Javenese’ or ‘other’.^34^

**Appendix 3**

**Estimation of sodium intake in the HELIUS study**

Four ethnic-specific FFQs to measure food intake were developed for the HELIUS study by Beukers et al, and were also validated for the respective populations.^35^ The FFQs collect information about the frequency and the amount of intake of approximately 200 food items in the previous 4 weeks. Based on data in the Dutch Food Composition Table 2011, a nutrient database for each FFQ was constructed.^37^ Each individual food item in the questionnaire was linked to one of more foods from this composition table. If more individual food items of the FFQs are linked to one item of the composition table, a weighted contribution of each individual food item was calculated based on the frequency of consumption (as percentage consumption days). To estimate sodium intake, we used the Dutch Food Composition Database (NEVO, online version December 2021) to identify the sodium content (mg/100 gram food) for each individual food item. With the weighted contribution of each single food item to the food items of the composition table, a total sodium content of each food item in the HELIUS nutrient database could be constructed. Based on the reported intake of each food item (mg/day) we could estimate sodium intake. Participants with implausibly high or low calorie intakes (<800 kcal/day and >4000 kcal/day for men, <500 kcal/day and >3500 kcal/day for women), were excluded.^36^ After exclusion based on above criteria, the cohort was stratified into tertiles of estimated sodium intake. Due to possible inaccuracy of the FFQ in estimating sodium intake, only the lowest and highest tertiles were used for analysis (see table below).

| **Characteristics** | **All** | **Dutch** | **South-Asian Surinamese** | **African Surinamese** | **Turkish** | **Moroccan** |
| --- | --- | --- | --- | --- | --- | --- |
| *N* | 2239 | 362 | 522 | 296 | 452 | 607 |
| Median estimated sodium intake T1 (mmol/day) | 56.0  (45.7-65.2) | 59.3  (52.1-65.7) | 54.5  (44.4-63.2) | 55.5  (45.0-33.4) | 57.7  (44.5-66.0) | 55.2  (44.4-65.1) |
| Median estimated sodium intake T2 (mmol/day) | 86.8  (79.1-95.3) | 87.4  (78.1-95.1) | 87.9  (79.7-95.3) | 86.3  (79.2-93.4) | 89.6  (80.9-96.5) | 84.7  (78.5-94.6) |
| Median stimated sodium intake T3 (mmol/day) | 127.8  (114.5-150.3) | 128.1  (115.8-140.2) | 121.5  (111.8-138.5) | 123.5  (109.3-143.2) | 135.5  (116.9-159.5) | 128.6  (114.5-151.1) |

*The median estimated sodium intake per tertile stratified for ethnicity. Data are shown as median with interquartile range.*

**Supplemental references**

34. Snijder MB, Galenkamp H, Prins Met al. Cohort profile: the Healthy Life in an Urban Setting (HELIUS) study in Amsterdam, The Netherlands. BMJ open. 2017;7(12):e017873.

35. Beukers MH, Dekker LH, de Boer EJet al. Development of the HELIUS food frequency questionnaires: ethnic-specific questionnaires to assess the diet of a multiethnic population in The Netherlands. European Journal of Clinical Nutrition. 2015;69(5):579-84.

36. Willett W. Nutritional epidemiology: Oxford university press; 2012.

37. Dutch Food Composition Table 2011. The Hague National Institute for Public Health and the Netherlands Nutrition Centre; 2011.

**Supplemental Tables**

**Table S1.** Glycosaminoglycan genes included in our analysis with corresponding chromosomal position (GrCh 37)

| **Gene** | **Chromosome** | **Chromosomal position (GrCh 37)** | |
| --- | --- | --- | --- |
|  |  | Start | End |
| ABCC5 | 3 | 183,637,722 | 183,735,803 |
| ACAN | 15 | 89,346,674 | 89,418,585 |
| AGRN | 1 | 955,503 | 991,496 |
| ARSB | 5 | 78,073,032 | 78,281,910 |
| B3GALT6 | 1 | 1,167,629 | 1,170,421 |
| B3GAT1 | 11 | 134,248,398 | 134,281,812 |
| B3GAT2 | 6 | 71,566,382 | 71,666,741 |
| B3GAT3 | 11 | 62,382,768 | 62,389,647 |
| B3GNT2 | 2 | 62,423,248 | 62,451,866 |
| B3GNT3 | 19 | 17,905,637 | 17,923,891 |
| B3GNT4 | 12 | 122,688,090 | 122,693,499 |
| B3GNT7 | 2 | 232,260,254 | 232,265,875 |
| B4GALT1 | 9 | 33,104,080 | 33,167,354 |
| B4GALT2 | 1 | 44,444,615 | 44,456,840 |
| B4GALT3 | 1 | 161,141,100 | 161,147,287 |
| B4GALT4 | 3 | 118,930,579 | 118,959,950 |
| B4GALT5 | 20 | 48,249,482 | 48,330,415 |
| B4GALT6 | 18 | 29,202,210 | 29,265,799 |
| B4GALT7 | 5 | 177,027,101 | 177,037,348 |
| B4GAT1 | 11 | 76,745,385 | 76,753,096 |
| BCAN | 1 | 156,611,182 | 156,629,324 |
| BGN | X | 152,760,397 | 152,775,012 |
| CD44 | 11 | 35,160,417 | 35,253,949 |
| CEMIP | 15 | 81,071,684 | 81,244,117 |
| CHP1 | 15 | 41,523,037 | 41,574,043 |
| CHPF | 2 | 220,403,669 | 220,408,509 |
| CHPF2 | 7 | 150,929,575 | 150,935,908 |
| CHST1 | 11 | 45,670,427 | 45,687,172 |
| CHST11 | 12 | 104,849,073 | 105,155,792 |
| CHST12 | 7 | 2,443,223 | 2,474,242 |
| CHST13 | 3 | 126,243,126 | 126,262,134 |
| CHST14 | 15 | 40,763,160 | 40,765,353 |
| CHST15 | 10 | 125,767,184 | 125,853,206 |
| CHST2 | 3 | 142,838,173 | 142,841,800 |
| CHST3 | 10 | 73,724,123 | 73,773,322 |
| CHST5 | 10 | 125,767,184 | 125,853,206 |
| CHST6 | 16 | 75,510,949 | 75,529,282 |
| CHST7 | X | 46,433,219 | 46,457,843 |
| CHST9 | 18 | 24,495,595 | 24,765,281 |
| CHSY1 | 15 | 101,715,928 | 101,792,137 |
| CHSY3 | 5 | 129,240,165 | 129,522,327 |
| CSGALNACT1 | 8 | 19,261,672 | 19,615,540 |
| CSGALNACT2 | 10 | 43,633,934 | 43,680,756 |
| CSPG4 | 15 | 75,966,663 | 76,005,189 |
| CSPG5 | 3 | 47,603,729 | 47,622,282 |
| DCN | 12 | 91,539,025 | 91,576,900 |
| DSE | 6 | 116,575,336 | 116,762,424 |
| DSEL | 18 | 65,173,819- | 65,184,217 |
| EXT1 | 8 | 118,806,729 | 119,124,092 |
| EXT2 | 11 | 44,117,099 | 44,266,979 |
| EXTL1 * | 1 | 26,346,375 | 26,362,955 |
| EXTL2 * | 1 | 101,337,943 | 101,361,554 |
| EXTL3 * | 8 | 28,457,986 | 28,613,116 |
| FAM20B * | 1 | 178,994,939 | 179,045,697 |
| FMOD | 1 | 203,309,756 | 203,320,617 |
| GALNS | 16 | 88,880,142 | 88,923,378 |
| GLB1 | 3 | 33,038,100 | 33,138,722 |
| GLB1L | 2 | 220,101,328 | 220,110,200 |
| GLCE | 15 | 69,452,923 | 69,564,556 |
| GNS | 12 | 65,107,225 | 65,153,227 |
| GPC1 | 2 | 241,375,088 | 241,407,493 |
| GPC2 | 7 | 99,767,229 | 99,774,995 |
| GPC3 | X | 132,669,773 | 133,119,922 |
| GPC4 | X | 132,434,131 | 132,549,518 |
| GPC5 | 13 | 92,050,929 | 93,519,490 |
| GPC6 | 13 | 93,879,095 | 95,059,655 |
| GUSB | 7 | 65,425,671 | 65,447,301 |
| HAS1 | 19 | 52,216,365 | 52,227,247 |
| HAS2 | 8 | 122,624,356 | 122,653,630 |
| HAS3 | 16 | 69,139,467 | 69,152,622 |
| HEXA | 15 | 72,635,775 | 72,668,817 |
| HEXB | 5 | 73,935,848 | 74,018,472 |
| HGSNAT | 8 | 42,995,556 | 43,057,998 |
| HMMR | 5 | 162,887,209 | 162,918,947 |
| HPSE | 4 | 84,213,614 | 84,256,306 |
| HPSE2 | 10 | 100,218,875 | 100,995,619 |
| HS2ST1 | 1 | 87,380,331 | 87,602,334 |
| HS3ST1 | 4 | 11,394,774 | 11,431,389 |
| HS3ST2 | 16 | 22,825,498 | 22,927,659 |
| HS3ST3A1 | 17 | 13,399,006 | 13,505,244 |
| HS3ST3B1 | 17 | 14,204,400 | 14,252,721 |
| HS3ST4 | 16 | 25,703,347 | 26,149,009 |
| HS3ST5 | 6 | 114,376,750 | 114,664,209 |
| HS3ST6 | 16 | 1,961,464 | 1,968,441 |
| HS6ST1 | 2 | 128,994,290 | 129,076,151 |
| HS6ST2 | X | 131,760,044 | 132,095,423 |
| HS6ST3 | 13 | 96,743,093 | 97,485,671 |
| HSPG2 | 1 | 22,148,738 | 22,263,790 |
| HYAL1 | 3 | 50,337,320 | 50,349,812 |
| HYAL2 | 3 | 50,355,221 | 50,360,337 |
| HYAL3 | 3 | 50,330,262 | 50,336,899 |
| IDS | X | 148,558,521 | 148,615,470 |
| IDUA | 4 | 980,785 | 998,316 |
| KERA | 12 | 91,444,268 | 91,451,760 |
| LUM | 12 | 91,496,406 | 91,505,608 |
| LYVE1 | 11 | 10,578,513 | 10,633,236 |
| NAGLU | 17 | 40,688,190 | 40,696,467 |
| NCAN | 19 | 19,322,782 | 19,363,042 |
| NDST1 | 5 | 149,865,381 | 149,937,773 |
| NDST2 | 10 | 75,561,669 | 75,571,589 |
| NDST3 | 4 | 118,954,773 | 119,179,803 |
| NDST4 | 4 | 115,748,919 | 116,035,032 |
| OGN | 9 | 95,146,249 | 95,166,978 |
| OMD | 9 | 95,176,527 | 95,186,743 |
| PAPSS1 | 4 | 108,511,433 | 108,641,608 |
| PAPSS2 | 10 | 89,419,370 | 89,507,462 |
| PRELP | 1 | 203,444,956 | 203,460,480 |
| SDC1 | 2 | 20,400,558 | 20,425,194 |
| SDC2 | 8 | 97,505,579 | 97,624,000 |
| SDC3 | 1 | 31,342,314 | 31,381,608 |
| SDC4 | 20 | 43,953,928 | 43,977,064 |
| SGSH | 17 | 78,180,515- | 78,194,722 |
| SLC26A1 | 4 | 972,861 | 987,228 |
| SLC26A2 | 5 | 149,340,300 | 149,373,018 |
| SLC35B2 | 6 | 44,221,833 | 44,225,291 |
| SLC35B3 | 6 | 8,413,301 | 8,435,794 |
| SLC35D2 | 9 | 99,082,988 | 99,145,992 |
| SLC9A1 | 1 | 27,425,306 | 27,493,472 |
| ST3GAL1 | 8 | 134,467,091 | 134,584,183 |
| ST3GAL2 | 16 | 70,413,338 | 70,473,140 |
| ST3GAL3 | 1 | 44,171,495 | 44,396,831 |
| ST3GAL4 | 11 | 126,225,535 | 126,310,239 |
| ST3GAL6 | 3 | 98,451,080 | 98,540,045 |
| STAB2 | 12 | 103,981,051 | 104,160,505 |
| SULF1 * | 8 | 70,378,859 | 70,573,150 |
| SULF2 * | 20 | 46,285,092 | 46,415,360 |
| UST | 6 | 149,068,464 | 149,398,126 |
| VCAN | 5 | 82,767,284 | 82,878,122 |
| XYLT1 | 16 | 17,195,626 | 17,564,738 |
| XYLT2 | 17 | 48,423,453 | 48,440,499 |

*Genes marked with * are included based on literature research. Other genes are extracted from Reactome®.*

**Table S2.**  Significant top SNPs for both formulas in EPIC Norfolk and their corresponding gene regions

| **BP trait** | **rsID** | **Chr** | **Pos** | **GAG- Gene region** | **A1** | **A2** | **MAF** | **β** | **SE** | **Kawasaki**  **FDR -**  **P value** | **Tanaka**  **FDR –**  **P value** |
| --- | --- | --- | --- | --- | --- | --- | --- | --- | --- | --- | --- |
| SBP | rs9481435 | 6 | 114574112 | HS3ST5 | G | A | 0.019 | -0.044 | 0.009 | 0.012 | 0.015 |
|  | rs56843725 | 4 | 119126987 | NDST3 | I | D | 0.140 | 0.016 | 0.004 | 0.034 | 0.041 |
|  | rs12368421 | 12 | 93373503 | GPC5 | C | T | 0.015 | 0.044 | 0.010 | 0.034 | 0.029 |
| DBP | rs114568494 | 1 | 22241660 | HSPG2 | G | A | 0.03 | 0.022 | 0.006 | 0.026 | 0.025 |
|  | rs6550188 | 3 | 33036135 | GLB1 | A | G | 0.25 | 0.008 | 0.002 | 0.014 | 0.025 |
|  | rs67580739 | 4 | 11411054 | HS3ST1 | G | T | 0.18 | -0.008 | 0.002 | 0.023 | 0.014 |
|  | rs112269249 | 4 | 119125452 | NDST3 | A | T | 0.27 | 0.011 | 0.002 | <0.001 | 0.001 |
|  | rs570975903 | 5 | 73926989 | HEXB | I | D | 0.13 | -0.011 | 0.002 | 0.004 | 0.002 |
|  | rs72070125 | 6 | 149215962 | UST | D | I | 0.39 | -0.008 | 0.002 | 0.018 | 0.046 |
|  | rs4738003 | 8 | 70542552 | SULF1 | C | T | 0.46 | 0.006 | 0.002 | 0.042 | 0.038 |
|  | rs2978028 | 8 | 134525141 | ST3GAL1 | A | T | 0.22 | 0.008 | 0.002 | 0.027 | 0.038 |
|  | rs3088003 | 12 | 92539460 | GPC5 | C | T | 0.11 | -0.011 | 0.003 | 0.023 | 0.004 |
|  | rs9516683 | 13 | 97052569 | HS6ST3 | A | G | 0.38 | 0.007 | 0.002 | 0.033 | 0.027 |
| MAP | rs114568494 | 1 | 22241660 | HSPG2 | G | A | 0.03 | 0.026 | 0.006 | 0.020 | 0.035 |
|  | rs11715480 | 3 | 33028828 | GLB1 | G | C | 0.27 | -0.008 | 0.002 | 0.024 | 0.035 |
|  | rs67580739 | 4 | 11411054 | HS3ST1 | G | T | 0.23 | -0.009 | 0.002 | 0.025 | 0.028 |
|  | rs112269249 | 4 | 119125452 | NDST3 | A | T | 0.27 | 0.011 | 0.002 | 0.007 | 0.003 |
|  | rs570975903 | 5 | 73926989 | HEXB | I | D | 0.13 | -0.013 | 0.003 | 0.011 | 0.006 |
|  | rs9488347 | 6 | 114563906 | HS3ST5 | A | C | 0.019 | -0.028 | 0.007 | 0.013 | 0.028 |
|  | rs72070125 | 6 | 149215962 | UST | D | I | 0.39 | -0.009 | 0.002 | 0.020 | 0.043 |
|  | rs5892204 | 8 | 70482148 | SULF1 | I | D | 0.36 | -0.007 | 0.002 | 0.043 | 0.034 |
|  | rs2978026 | 8 | 134524536 | ST3GAL1 | C | T | 0.22 | 0.009 | 0.002 | 0.023 | 0.038 |
|  | rs74527276 | 12 | 92290429 | GPC5 | T | C | 0.09 | -0.013 | -0.003 | 0.016 | 0.023 |
|  | rs181508522 | 17 | 78187811 | SGSH | T | C | 0.02 | -0.030 | 0.008 | 0.031 | 0.038 |
|  | rs6125107 | 20 | 46392496 | SULF2 | T | C | 0.34 | 0.007 | 0.002 | 0.046 | 0.038 |

*SNP, single nucleotide polymorphism. CHR, chromosome. POS, position based GRCh37. A1, allele 1. A2, allele 2. MAF, Minor allele frequency. β, for the interaction term with the Kawasaki formula. SE, standard error for the β-coefficient of the interaction term with the Kawasaki formula. FDR, false-rate-discovery.*

**Table S3.**  Significant SNPs for MAP from meta-analysis combining EPIC Norfolk and UK biobank

| **rsID** | **Chr** | **Pos** | **A1** | **A2** | **MAF** | **Direction** | **Kawasaki FDR – P value** | **Tanaka FDR – p value** |
| --- | --- | --- | --- | --- | --- | --- | --- | --- |
| rs2892799 | 4 | 119124720 | T | C | 0.183 | ++ | 0.034 | 0.039 |
| rs2140475 | 4 | 119121473 | T | C | 0.183 | ++ | 0.034 | 0.039 |
| rs112269249 | 4 | 119125452 | A | T | 0.269 | ++ | 0.036 | 0.039 |
| rs674514 | 4 | 119184569 | C | T | 0.187 | -- | 0.036 | 0.039 |
| rs35433749 | 4 | 119049511 | A | G | 0.348 | ++ | 0.036 | 0.039 |
| rs6820367 | 4 | 119092610 | A | C | 0.349 | ++ | 0.036 | 0.039 |
| rs12501122 | 4 | 119048385 | G | T | 0.348 | -- | 0.036 | 0.039 |
| rs6534089 | 4 | 119081987 | G | A | 0.354 | -- | 0.036 | 0.039 |
| rs17593919 | 4 | 119060552 | G | A | 0.349 | -- | 0.036 | 0.039 |
| rs4834668 | 4 | 119105539 | C | T | 0.348 | -- | 0.036 | 0.039 |
| rs34548920 | 4 | 119106609 | T | C | 0.348 | ++ | 0.036 | 0.039 |
| rs56843725 | 4 | 119126987 | I | D | 0.140 | -- | 0.036 | 0.039 |
| rs4240320 | 4 | 119114424 | C | T | 0.348 | -- | 0.036 | 0.039 |
| rs12642204 | 4 | 119064257 | T | C | 0.349 | ++ | 0.036 | 0.039 |
| rs34165051 | 4 | 119069869 | G | C | 0.349 | -- | 0.036 | 0.039 |
| rs4834667 | 4 | 119066243 | T | C | 0.349 | ++ | 0.036 | 0.039 |
| rs13119835 | 4 | 119060838 | C | T | 0.349 | -- | 0.036 | 0.039 |
| rs4318721 | 4 | 119039932 | A | G | 0.347 | ++ | 0.036 | 0.039 |
| rs13123722 | 4 | 119073293 | G | A | 0.348 | -- | 0.036 | 0.039 |
| rs13149888 | 4 | 119074927 | C | T | 0.349 | -- | 0.036 | 0.039 |
| rs4833565 | 4 | 119117260 | T | C | 0.348 | ++ | 0.036 | 0.039 |
| rs12504529 | 4 | 119109714 | A | G | 0.348 | ++ | 0.036 | 0.039 |
| rs35760279 | 4 | 119123242 | G | A | 0.348 | -- | 0.036 | 0.039 |
| rs4833564 | 4 | 119079639 | A | G | 0.349 | ++ | 0.036 | 0.039 |
| rs35235550 | 4 | 119087151 | A | G | 0.348 | ++ | 0.036 | 0.039 |
| rs11322429 | 4 | 119042839 | I | D | 0.349 | -- | 0.036 | 0.039 |
| rs4240319 | 4 | 119079882 | C | A | 0.349 | -- | 0.036 | 0.039 |
| rs12506091 | 4 | 119088127 | G | C | 0.348 | -- | 0.036 | 0.039 |
| rs1916686 | 4 | 119119213 | A | G | 0.348 | ++ | 0.036 | 0.039 |
| rs201324921 | 4 | 119070647 | G | T | 0.348 | -- | 0.036 | 0.039 |
| rs202075542 | 4 | 119070653 | T | G | 0.347 | ++ | 0.036 | 0.039 |
| rs34826732 | 4 | 119051187 | A | G | 0.348 | ++ | 0.036 | 0.039 |
| rs4438821 | 4 | 118949069 | T | C | 0.156 | ++ | 0.036 | 0.039 |
| rs35524586 | 4 | 119119882 | I | D | 0.327 | ++ | 0.036 | 0.039 |
| rs6851494 | 4 | 119054788 | G | C | 0.189 | -- | 0.036 | 0.039 |
| rs10012787 | 4 | 118945044 | A | T | 0.155 | ++ | 0.036 | 0.039 |
| rs10440342 | 4 | 118945478 | A | G | 0.155 | ++ | 0.036 | 0.039 |
| rs10440399 | 4 | 118945522 | T | C | 0.155 | ++ | 0.036 | 0.039 |
| rs7688055 | 4 | 118946388 | A | C | 0.155 | ++ | 0.036 | 0.039 |
| rs6534074 | 4 | 118948432 | C | T | 0.155 | -- | 0.036 | 0.039 |
| rs1319061 | 4 | 118948608 | T | A | 0.155 | -- | 0.036 | 0.039 |
| rs2389491 | 4 | 118947929 | T | A | 0.155 | -- | 0.036 | 0.039 |
| rs76631779 | 4 | 118948225 | A | C | 0.156 | ++ | 0.036 | 0.039 |
| rs2389492 | 4 | 118949470 | T | C | 0.155 | ++ | 0.036 | 0.039 |
| rs2291416 | 4 | 118949653 | G | A | 0.155 | -- | 0.036 | 0.039 |
| rs2254218 | 4 | 118949828 | T | C | 0.155 | ++ | 0.036 | 0.039 |
| rs2389493 | 4 | 118949490 | T | C | 0.155 | ++ | 0.036 | 0.039 |
| rs2254215 | 4 | 118949878 | A | G | 0.155 | ++ | 0.036 | 0.039 |
| rs139967941 | 4 | 118946508 | I | D | 0.155 | -- | 0.036 | 0.039 |
| rs10000063 | 4 | 118948240 | C | T | 0.155 | -- | 0.036 | 0.039 |
| rs2254204 | 4 | 118950060 | T | C | 0.155 | ++ | 0.036 | 0.039 |
| rs2389494 | 4 | 118951262 | T | C | 0.155 | ++ | 0.036 | 0.039 |
| rs6534077 | 4 | 118953737 | T | C | 0.155 | ++ | 0.036 | 0.039 |
| rs6534075 | 4 | 118952114 | C | A | 0.155 | -- | 0.036 | 0.039 |
| rs6534078 | 4 | 118953901 | A | G | 0.155 | ++ | 0.036 | 0.039 |
| rs7671497 | 4 | 118953935 | T | G | 0.155 | ++ | 0.036 | 0.039 |
| rs9999038 | 4 | 118960427 | A | G | 0.155 | ++ | 0.036 | 0.039 |
| rs72524667 | 4 | 118968077 | D | I | 0.155 | ++ | 0.036 | 0.039 |
| rs2389495 | 4 | 118960142 | G | A | 0.155 | -- | 0.036 | 0.039 |
| rs7657704 | 4 | 118966732 | C | T | 0.155 | -- | 0.036 | 0.039 |
| rs2389498 | 4 | 118967536 | T | C | 0.155 | ++ | 0.036 | 0.039 |
| rs33979057 | 4 | 118945794 | I | D | 0.154 | -- | 0.036 | 0.041 |
| rs12641173 | 4 | 119038472 | T | A | 0.327 | -- | 0.036 | 0.041 |
| rs67374916 | 4 | 119037194 | I | D | 0.328 | -- | 0.036 | 0.041 |
| rs6820851 | 4 | 119016217 | T | G | 0.327 | ++ | 0.036 | 0.041 |
| rs35294396 | 4 | 119030052 | I | D | 0.328 | -- | 0.036 | 0.041 |
| rs10546006 | 4 | 118951176 | I | D | 0.158 | -- | 0.042 | 0.041 |
| rs6534084 | 4 | 119037650 | C | T | 0.327 | -- | 0.036 | 0.041 |
| rs67248472 | 4 | 119022523 | D | I | 0.178 | ++ | 0.038 | 0.041 |
| rs4544776 | 4 | 119033124 | C | T | 0.327 | -- | 0.036 | 0.041 |
| rs11098426 | 4 | 119015832 | C | A | 0.327 | -- | 0.036 | 0.041 |
| rs11299569 | 4 | 118984344 | I | D | 0.111 | -- | 0.042 | 0.041 |
| rs4428355 | 4 | 119020848 | G | A | 0.327 | -- | 0.038 | 0.041 |
| rs17692850 | 4 | 119022268 | A | G | 0.327 | ++ | 0.038 | 0.041 |
| rs4240315 | 4 | 119024014 | T | C | 0.327 | ++ | 0.038 | 0.041 |
| rs71627753 | 4 | 119028262 | A | C | 0.131 | ++ | 0.042 | 0.041 |
| rs11384076 | 4 | 118980809 | D | I | 0.134 | ++ | 0.042 | 0.041 |
| rs2892787 | 4 | 118991092 | A | G | 0.133 | ++ | 0.042 | 0.041 |
| rs13135646 | 4 | 118992195 | G | C | 0.133 | -- | 0.042 | 0.041 |
| rs13135500 | 4 | 118992273 | C | T | 0.133 | -- | 0.042 | 0.041 |
| rs11722962 | 4 | 118992398 | T | C | 0.133 | ++ | 0.042 | 0.041 |
| rs11098422 | 4 | 118989467 | G | A | 0.133 | -- | 0.042 | 0.041 |
| rs7656160 | 4 | 118973250 | C | A | 0.155 | -- | 0.042 | 0.041 |
| rs4558930 | 4 | 118991191 | C | T | 0.133 | -- | 0.042 | 0.041 |
| rs2389509 | 4 | 118985259 | T | G | 0.133 | ++ | 0.042 | 0.041 |
| rs13137083 | 4 | 118985608 | G | C | 0.133 | -- | 0.042 | 0.041 |
| rs6816778 | 4 | 118978908 | A | C | 0.133 | ++ | 0.042 | 0.041 |
| rs6818042 | 4 | 118979504 | A | G | 0.133 | ++ | 0.042 | 0.041 |
| rs11730611 | 4 | 118982899 | G | A | 0.133 | -- | 0.042 | 0.041 |
| rs2892785 | 4 | 118984212 | G | A | 0.133 | -- | 0.042 | 0.041 |
| rs2892786 | 4 | 118984635 | G | T | 0.133 | -- | 0.042 | 0.041 |
| rs10020473 | 4 | 118995395 | C | T | 0.133 | -- | 0.042 | 0.041 |
| rs13125397 | 4 | 118996105 | A | G | 0.133 | ++ | 0.042 | 0.041 |
| rs11733562 | 4 | 118996226 | G | A | 0.133 | -- | 0.042 | 0.041 |
| rs10027013 | 4 | 118997594 | C | T | 0.133 | -- | 0.042 | 0.041 |
| rs2389512 | 4 | 118997822 | C | T | 0.133 | -- | 0.042 | 0.041 |
| rs13139875 | 4 | 118997985 | C | T | 0.133 | -- | 0.042 | 0.041 |
| rs1994384 | 4 | 118998778 | A | C | 0.133 | ++ | 0.042 | 0.041 |
| rs4834656 | 4 | 118999480 | G | A | 0.133 | -- | 0.042 | 0.041 |
| rs7688063 | 4 | 118976492 | A | G | 0.133 | ++ | 0.042 | 0.041 |
| rs12505642 | 4 | 118982231 | C | G | 0.133 | ++ | 0.042 | 0.041 |
| rs2389500 | 4 | 118976828 | A | C | 0.133 | ++ | 0.042 | 0.041 |
| rs4240313 | 4 | 118977621 | A | C | 0.133 | ++ | 0.042 | 0.041 |
| rs4240314 | 4 | 118977688 | T | C | 0.133 | ++ | 0.042 | 0.041 |
| rs2389501 | 4 | 118977941 | A | G | 0.133 | ++ | 0.042 | 0.041 |
| rs2389503 | 4 | 118978057 | T | C | 0.133 | ++ | 0.042 | 0.041 |
| rs11098421 | 4 | 118978766 | T | A | 0.133 | -- | 0.042 | 0.041 |
| rs7688443 | 4 | 118976620 | C | T | 0.133 | -- | 0.042 | 0.041 |
| rs7687849 | 4 | 118976318 | C | T | 0.133 | -- | 0.042 | 0.041 |
| rs1872771 | 4 | 118977241 | C | T | 0.133 | -- | 0.042 | 0.041 |
| rs13104129 | 4 | 119000491 | G | C | 0.133 | -- | 0.042 | 0.042 |

*SNP, single nucleotide polymorphism. CHR, chromosome. POS, position based GRCh37. A1, allele 1. A2, allele 2. MAF, Minor allele frequency. FDR, false-rate-discovery.*

**Table S4.**  Significant SNPs for SBP from meta-analysis combining EPIC Norfolk and UK biobank

| **rsID** | **Chr** | **Pos** | **A1** | **A2** | **MAF** | **Direction** | **Kawasaki FDR –**  **P value** | **Tanaka FDR –**  **p value** |
| --- | --- | --- | --- | --- | --- | --- | --- | --- |
| rs56843725 | 4 | 119126987 | I | D | 0.140 | -- | 0.027 | 0.042 |
| rs4438821 | 4 | 118949069 | T | C | 0.156 | ++ | 0.027 | 0.042 |
| rs10012787 | 4 | 118945044 | A | T | 0.155 | ++ | 0.027 | 0.042 |
| rs10440342 | 4 | 118945478 | A | G | 0.155 | ++ | 0.027 | 0.042 |
| rs10440399 | 4 | 118945522 | T | C | 0.155 | ++ | 0.027 | 0.042 |
| rs7688055 | 4 | 118946388 | A | C | 0.155 | ++ | 0.027 | 0.042 |
| rs2389491 | 4 | 118947929 | T | A | 0.155 | -- | 0.027 | 0.042 |
| rs6534074 | 4 | 118948432 | C | T | 0.155 | -- | 0.027 | 0.042 |
| rs1319061 | 4 | 118948608 | T | A | 0.155 | -- | 0.027 | 0.042 |
| rs2389492 | 4 | 118949470 | T | C | 0.155 | ++ | 0.027 | 0.042 |
| rs2291416 | 4 | 118949653 | G | A | 0.155 | -- | 0.027 | 0.042 |
| rs139967941 | 4 | 118946508 | I | D | 0.155 | -- | 0.027 | 0.042 |
| rs2254218 | 4 | 118949828 | T | C | 0.155 | ++ | 0.027 | 0.042 |
| rs2389493 | 4 | 118949490 | T | C | 0.155 | ++ | 0.027 | 0.042 |
| rs72524667 | 4 | 118968077 | D | I | 0.155 | ++ | 0.027 | 0.042 |
| rs2254215 | 4 | 118949878 | A | G | 0.155 | ++ | 0.027 | 0.042 |
| rs76631779 | 4 | 118948225 | A | C | 0.156 | ++ | 0.027 | 0.042 |
| rs10000063 | 4 | 118948240 | C | T | 0.155 | -- | 0.027 | 0.042 |
| rs2254204 | 4 | 118950060 | T | C | 0.155 | ++ | 0.027 | 0.042 |
| rs2389494 | 4 | 118951262 | T | C | 0.155 | ++ | 0.027 | 0.042 |
| rs6534078 | 4 | 118953901 | A | G | 0.155 | ++ | 0.027 | 0.042 |
| rs7671497 | 4 | 118953935 | T | G | 0.155 | ++ | 0.027 | 0.042 |
| rs6534077 | 4 | 118953737 | T | C | 0.155 | ++ | 0.027 | 0.042 |
| rs6534075 | 4 | 118952114 | C | A | 0.155 | -- | 0.027 | 0.042 |
| rs9999038 | 4 | 118960427 | A | G | 0.155 | ++ | 0.027 | 0.042 |
| rs2389495 | 4 | 118960142 | G | A | 0.155 | -- | 0.027 | 0.042 |
| rs7657704 | 4 | 118966732 | C | T | 0.155 | -- | 0.027 | 0.042 |
| rs2389498 | 4 | 118967536 | T | C | 0.155 | ++ | 0.027 | 0.042 |
| rs33979057 | 4 | 118945794 | I | D | 0.154 | -- | 0.027 | 0.043 |
| rs9654628 | 6 | 114572043 | A | G | 0.019 | -- | 0.027 | 0.048 |
| rs7656160 | 4 | 118973250 | C | A | 0.155 | -- | 0.027 | 0.048 |
| rs73542323 | 6 | 114574342 | C | T | 0.019 | ++ | 0.038 | 0.048 |
| rs9481435 | 6 | 114574112 | G | A | 0.019 | ++ | 0.027 | 0.048 |
| rs9654629 | 6 | 114572171 | A | G | 0.019 | -- | 0.027 | 0.048 |
| rs9488349 | 6 | 114566884 | A | C | 0.019 | -- | 0.027 | 0.048 |
| rs9488350 | 6 | 114566945 | A | C | 0.019 | -- | 0.027 | 0.048 |
| rs9488347 | 6 | 114563906 | A | C | 0.019 | -- | 0.027 | 0.048 |
| rs9488348 | 6 | 114563933 | C | T | 0.019 | ++ | 0.027 | 0.048 |
| rs202129796 | 6 | 114565021 | D | I | 0.019 | -- | 0.027 | 0.048 |
| rs7757437 | 6 | 114593089 | T | C | 0.017 | -- | 0.027 | 0.048 |
| rs7775945 | 6 | 114593521 | A | G | 0.017 | -- | 0.027 | 0.048 |
| rs9488362 | 6 | 114590524 | A | G | 0.017 | -- | 0.027 | 0.048 |
| rs9488361 | 6 | 114587660 | T | G | 0.017 | -- | 0.027 | 0.048 |
| rs9488352 | 6 | 114577180 | C | T | 0.017 | ++ | 0.027 | 0.048 |
| rs73542342 | 6 | 114585822 | T | C | 0.017 | -- | 0.027 | 0.048 |
| rs9481438 | 6 | 114580909 | T | C | 0.017 | -- | 0.027 | 0.048 |
| rs9488357 | 6 | 114581316 | G | A | 0.017 | ++ | 0.027 | 0.048 |
| rs9488356 | 6 | 114581202 | A | G | 0.017 | -- | 0.027 | 0.048 |
| rs9488354 | 6 | 114578019 | C | T | 0.017 | ++ | 0.027 | 0.048 |
| rs112013055 | 6 | 114556218 | C | T | 0.017 | ++ | 0.027 | 0.048 |
| rs9481437 | 6 | 114579963 | A | T | 0.017 | -- | 0.027 | 0.048 |

*SNP, single nucleotide polymorphism. CHR, chromosome. POS, position based GRCh37. A1, allele 1. A2, allele 2. MAF, Minor allele frequency. FDR, false-rate-discovery.*

**Table S5.**  Baseline characteristics of the participants of the HELIUS cohort stratified for ethnicity

|  | **All** | **Dutch** | **South-Asian Surinamese** | **African Surinamese** | **Turkish** | **Moroccan** |
| --- | --- | --- | --- | --- | --- | --- |
| N | 2239 | 362 | 522 | 296 | 452 | 607 |
| Male, % | 42.6 | 50.6 | 40.0 | 37.8 | 47.3 | 38.9 |
| Age, year | 47 (12) | 54 (11) | 49 (12) | 53 (9) | 42 (11) | 42 (12) |
| BMI, kg/m^2^ | 27.2 (4.9) | 25.2 (3.8) | 26.5 (4.5) | 28.2 (5.3) | 28.3 (5.0) | 27.8 (5.0) |
| SBP, mmHg | 128.5 (17.8) | 129.8 (17.0) | 130.9 (18.8) | 136.6 (18.3) | 125.3 (15.9) | 124.2 (16.8) |
| DBP, mmHg | 79.1 (10.5) | 78.8 (10.3) | 80.7 (10.1) | 84.3 (9.9) | 78.6 (10.3) | 75.8 (10.1) |
| Hypertension, % | 35.7 | 38.1 | 45.2 | 59.8 | 26.3 | 21.3 |
| Antihypertensive medication, % | 18.1 | 18.2 | 26.4 | 33.4 | 11.1 | 8.7 |
| eGFR, CKD-epi ml/min/1.73m^2 a^ | 100.7  [88.6-110.9] | 88.6  [77.8-98.3] | 94.7  [83.7-104.0] | 98.2  [87.5-110.2] | 105.9  [97.9-113.5] | 108.9  [99.1-117.8] |

Data are depicted as mean (SD) or median (IQR) if marked with ^a^. Hypertension is defined as SBP >140 or DBP >90 or use of anti-hypertensive drugs. Daily sodium consumption was estimated based on Food Frequency Questionnaires. SBP, systolic blood pressure. DBP, diastolic blood pressure. BMI, body mass index. eGFR, estimated glomerular filtration rate.

**Table S6.** Characteristics of study participants stratified for BP response after HSD

|  | **No BP increase (n=6)** | | | **BP increase (n=6)** | | | |
| --- | --- | --- | --- | --- | --- | --- | --- |
|  | **LSD** | **HSD** | **p** | | **LSD** | **HSD** | **p** |
| **Office BP** |  |  |  | |  |  |  |
| Supine systolic BP (mmHg) | 119.8 (4.2) | 117.6 (2.3) | 0.22 | | 114.9 (10.0) | 120.0 (7.7) | **0.01** |
| Supine diastolic BP (mmHg) | 57.9 (4.5) | 56.1 (2.6) | 0.24 | | 58.7 (6.7) | 58.7 (7.4) | 1.0 |
| Supine MAP (mmHg) | 78.5 (2.6) | 76.6 (2.2) | **0.04** | | 77.4 (6.6) | 79.1 (6.6) | **<0.01** |
| **Plasma** |  |  |  | |  |  |  |
| Sodium (mmol/L)^a^ | 138.0 [135.5-138.3] | 139.0 [138.8 – 140.5] | **0.03** | | 138.0 [136.8-139.0] | 141.0 [139.0-142.5] | 0.07 |
| Potassium (mmol/L) | 4.0 (0.4) | 3.9 (0.2) | 0.50 | | 3.9 (0.2) | 3.9 (0.1) | 0.58 |
| Osmolality (mOsm/kg)^a^ | 284.5 [281.0 – 287.3] | 288.0 [286.0-292.3] | **0.04** | | 285.5 [282.8-288.3] | 289.0 [287.5-293.0] | 0.14 |
| Bicarbonate (mmol/L) | 24.9 (2.4) | 25.6 (2.1) | 0.69 | | 27.0 (2.1) | 25.1 (1.0) | 0.09 |
| Chloride (mmol/L) | 99.3 (2.0) | 102.8 (2.2) | **<0.01** | | 99.8 (1.0) | 103.8 (1.7) | **<0.01** |
| Urea (mmol/L) | 5.1 (1.1) | 4.5 (0.8) | 0.05 | | 4.8 (0.6) | 5.2 (0.9) | 0.33 |
| **24h urine** |  |  |  | |  |  |  |
| Volume (ml/24h) | 1820.0 (752.0) | 1870.2 (629.7) | 0.85 | | 1584.2 (261.6) | 1947.5 (501.8) | 0.19 |
| Sodium (mmol/24h) | 20.8 (11.7) | 318.6 (109.9) | **<0.01** | | 17.5 (7.5) | 363.0 (102.8) | **<0.01** |
| Potassium (mmol/24h) | 101.9 (25.2) | 84.2 (10.5) | **<0.01** | | 73.5 (18.6) | 95.9 (27.0) | **<0.01** |
| Urea (mmol/24h) | 478.4 (75.2) | 510.5 (61.1) | **0.05** | | 340.3 (87.7) | 468.1 (104.3) | 0.33 |
| Chloride (mmol/24h)^a^ | 24.1 [18.6-25.7] | 284.7 [227.0-383.4] | **0.03** | | 25.6 [21.9-25.9] | 366.8 [307.1-468.2] | **0.04** |
| Osmolality(mOsm/kg)^a^ | 444.5 [320.0-721.5] | 740.0 [661.0-836.5] | 0.08 | | 367.5 [318.3 -367.5] | 664.5 [601.5-925.3] | **0.05** |
| Creatinine (mmol/24) | 16.9 (2.5) | 18.1 (1.9) | **<0.01** | | 14.7 (1.9) | 16.5 (2.9) | **<0.01** |

*Data are depicted as mean (SD) or as median (Q1-Q3) when marked with ^s^. Normal distributed data were compared with the paired t-test and non-normal distributed data (^s^) are tested with the Wilcoxon signed rank test. BP, blood pressure. MAP, mean arterial pressure.*

**Supplemental Figures:**

**
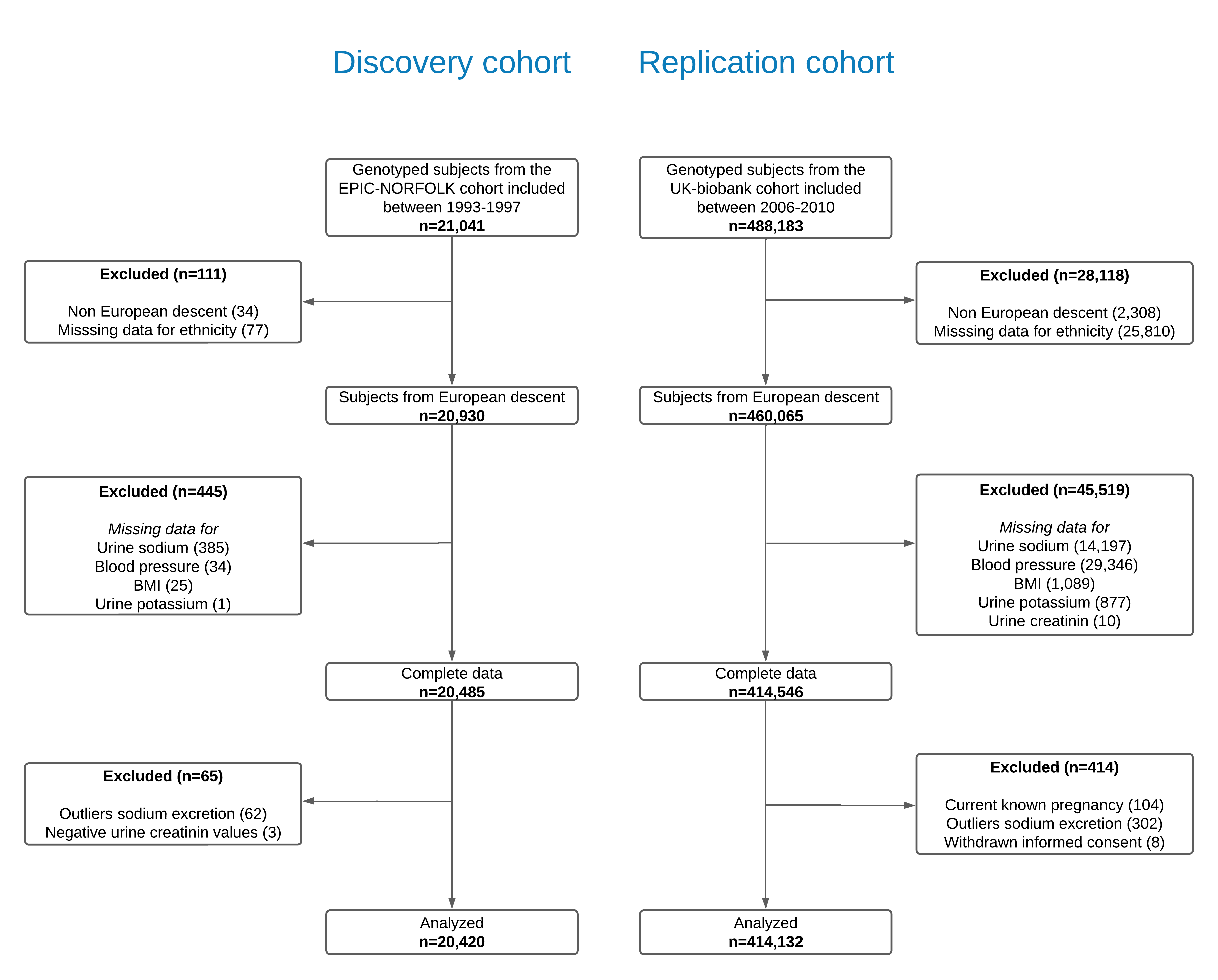
**

**Figure S1.** Flowchart of inclusion.

20,420 individuals from EPIC Norfolk and 414,132 individuals from UK Biobank were analyzed. Outliers in sodium excretion were based on the estimated sodium excretion obtained with the Kawasaki formula which is showed in Appendix 1. BMI, body mass index.


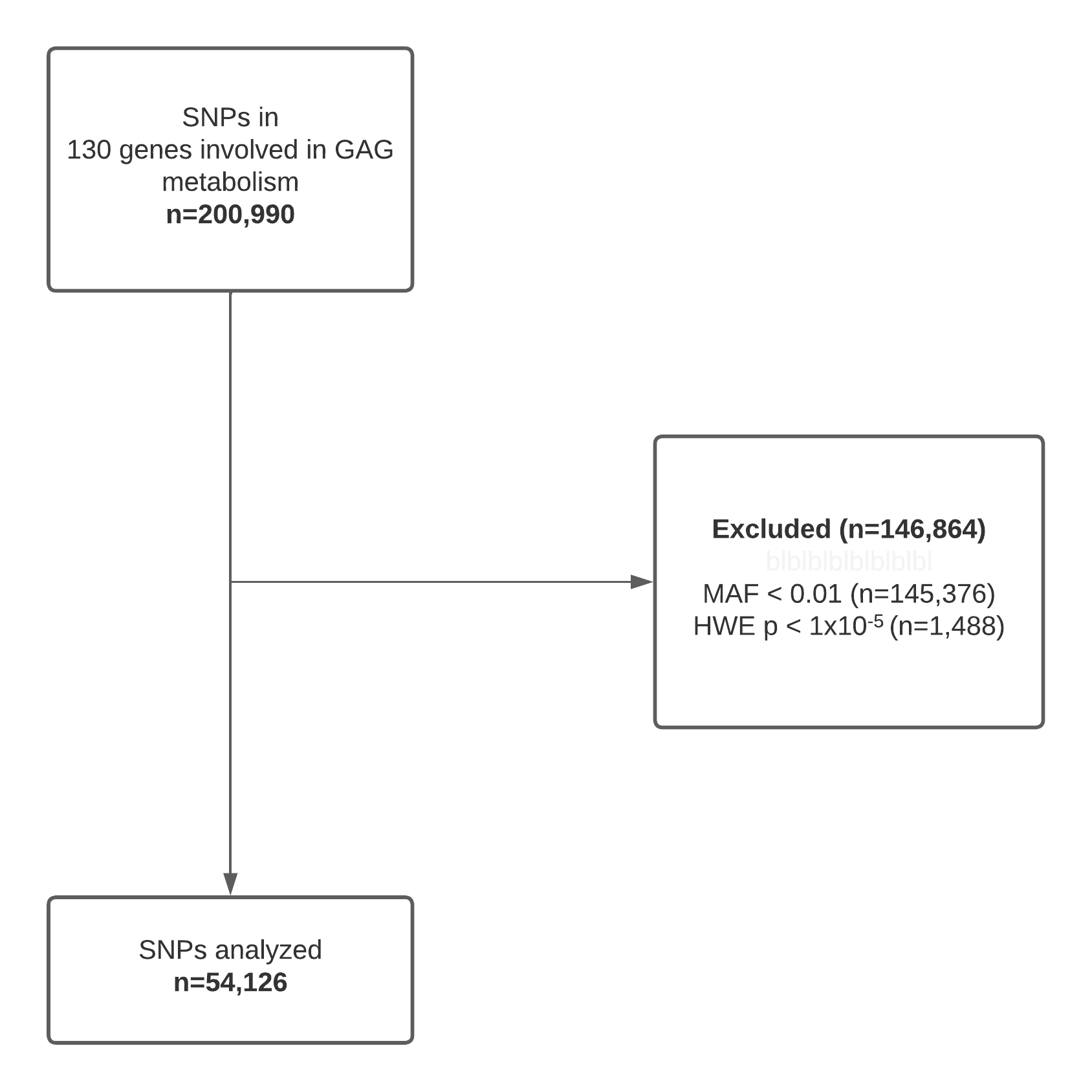


**Figure S2**. Flowchart of selected SNPs in EPIC Norfolk based on quality control

SNP, single nucleotide polymorphism. GAG, glycosaminoglycan. MAF, Minor Allele Frequency. HWE, Hardy Weinberg Equilibrium.


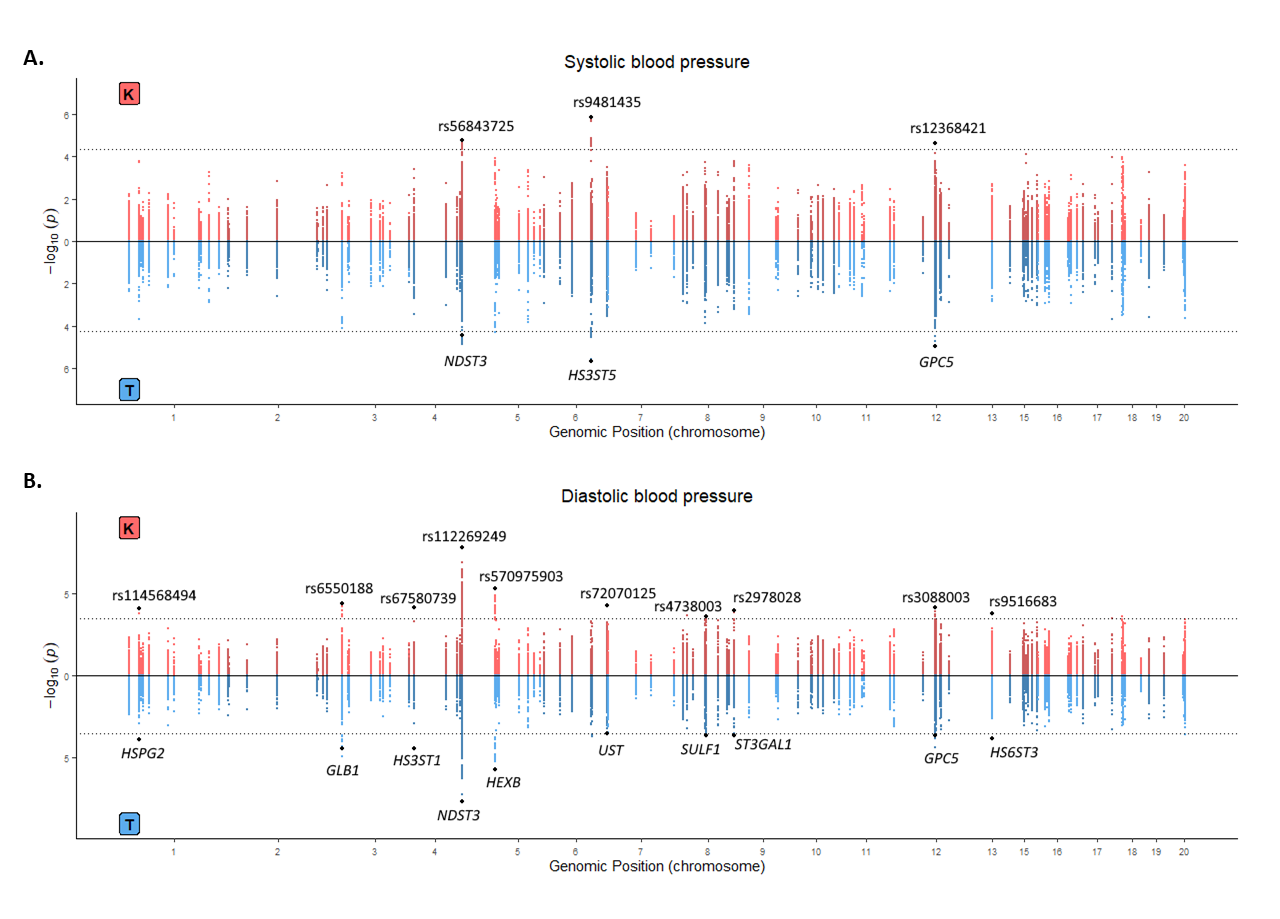


**Figure S3**. Miami plots for diastolic blood pressure results of EPIC Norfolk

The significance the interaction term of individual SNPs over the model without interaction term is visualized. The upper part of the graphs shows the significance for sodium intake estimated by the Kawasaki formula. The lower part of the graphs are for the results with estimated sodium intake with the Tanaka formula. SNP rsids as well as the corresponding glycosaminoglycan gene genomic region are visualized. The dotted line represents the FDR<0.05 significance level. K, Kawasaki. T, Tanaka

**
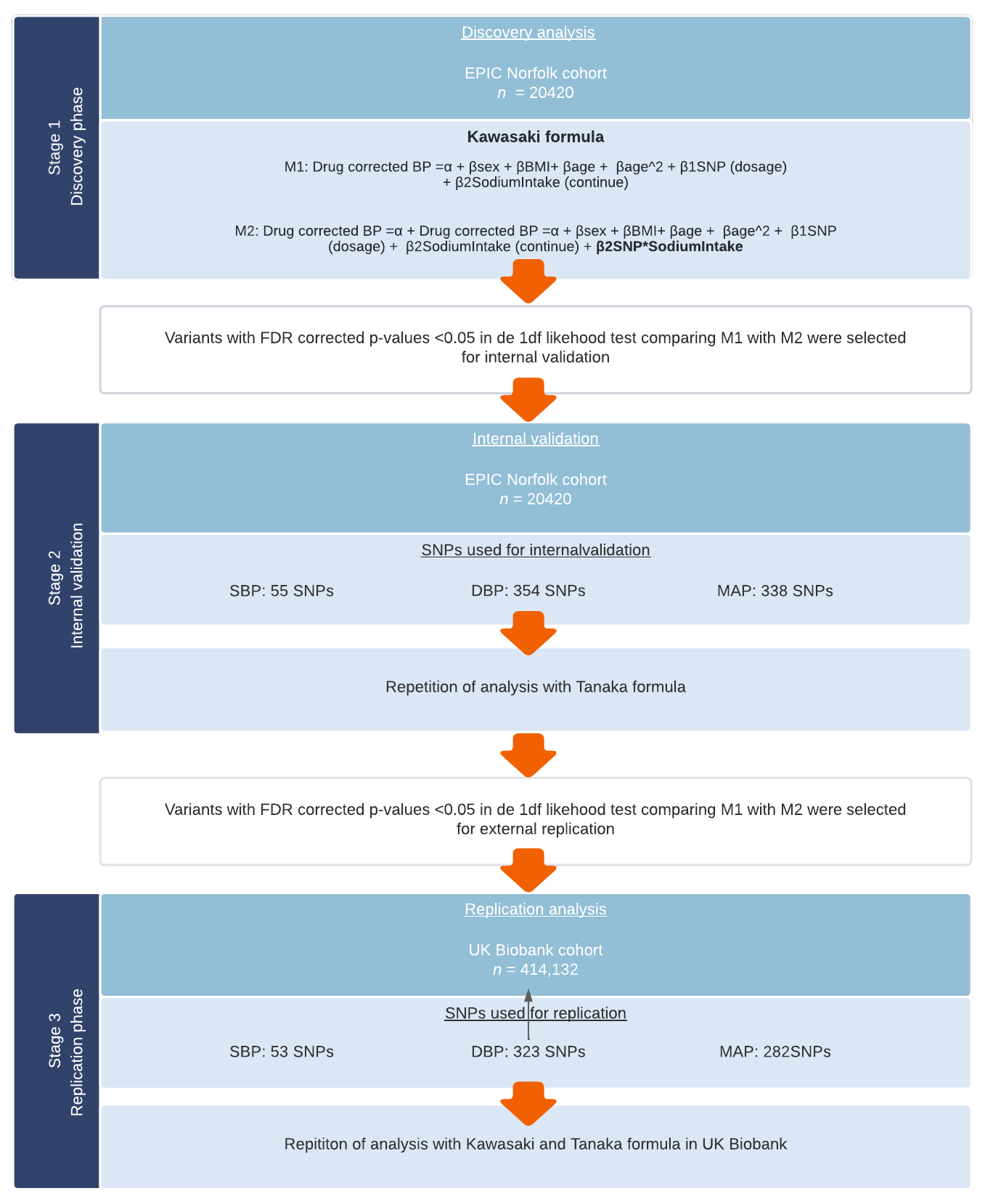
**

**Figure S4.** Flowchart of SNPs selected for replication in UK biobank

53 SNPs for SBP, 323 SNPs for DBP and 282 SNPs for MAP showed a significant interaction with estimated 24-hour sodium intake on BP phenotype with both sodium intake estimation formulas. These SNPs were replicated for their corresponding BP phenotype in UK Biobank. SBP, systolic blood pressure. DBP, diastolic blood pressure. M1, method 1 (without interaction). M2, method2 (with interaction term). FDR, false-rate-discovery. SNP, single nucleotide polymorphism.


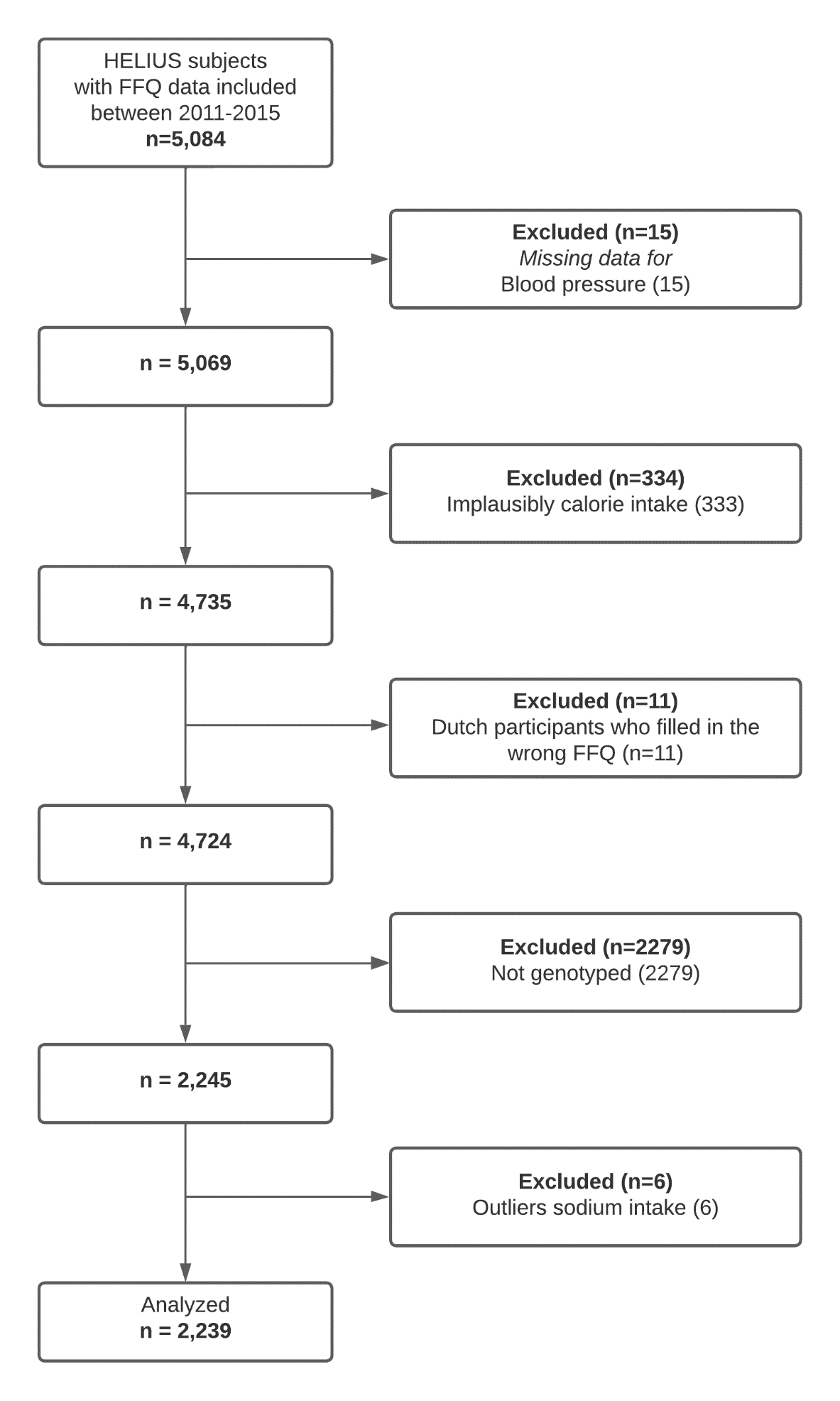


**Figure S5.** Flowchart for selection of participants in the HELIUS study. FFQ, food frequency questionnaire.

**
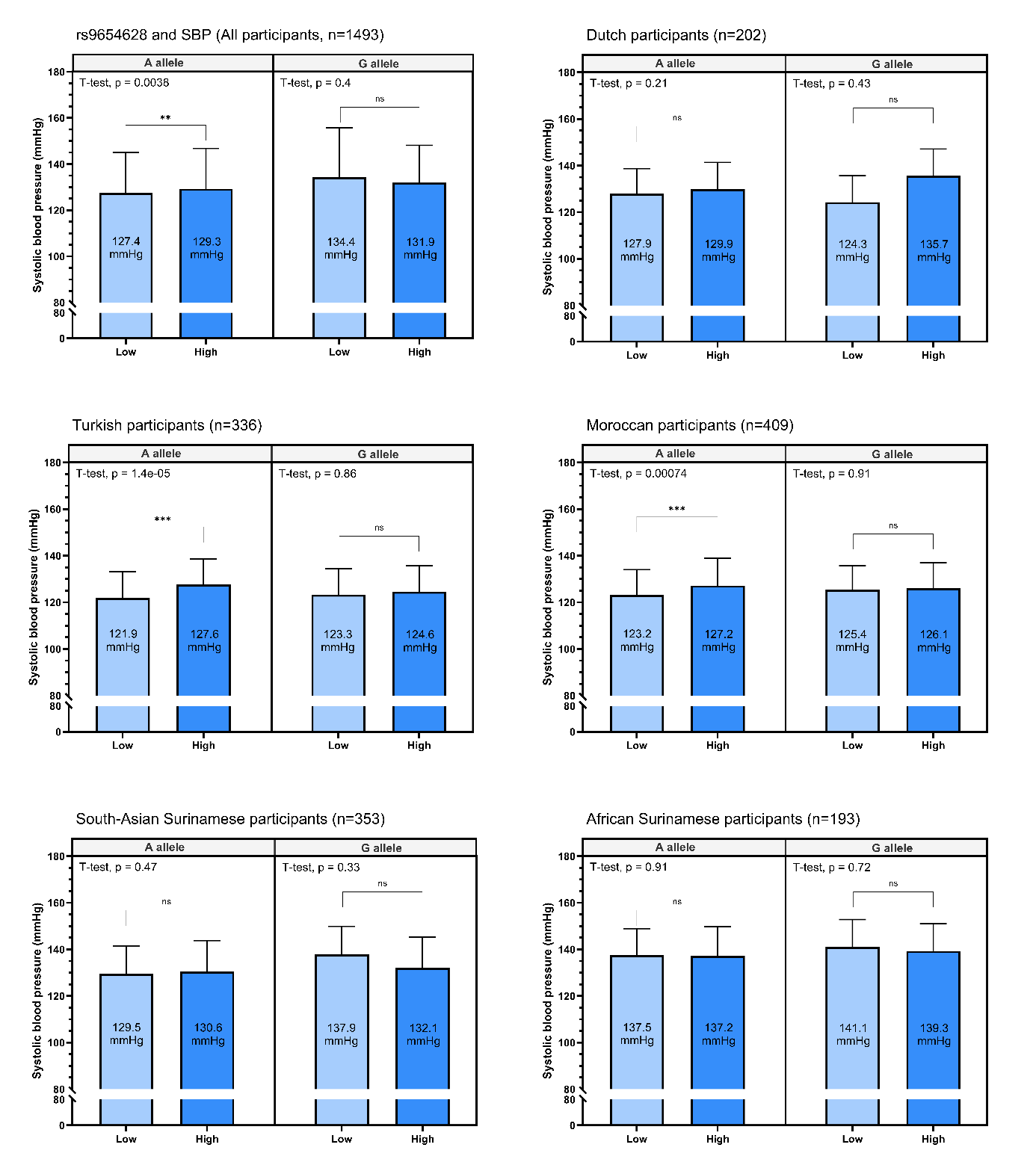
**

**Figure S6.** Systolic BP stratified for alleles of rs9654628 and sodium intake estimated with food frequency questionnaires for every ethnic group in the HELIUS population stratified for ethnicity

**
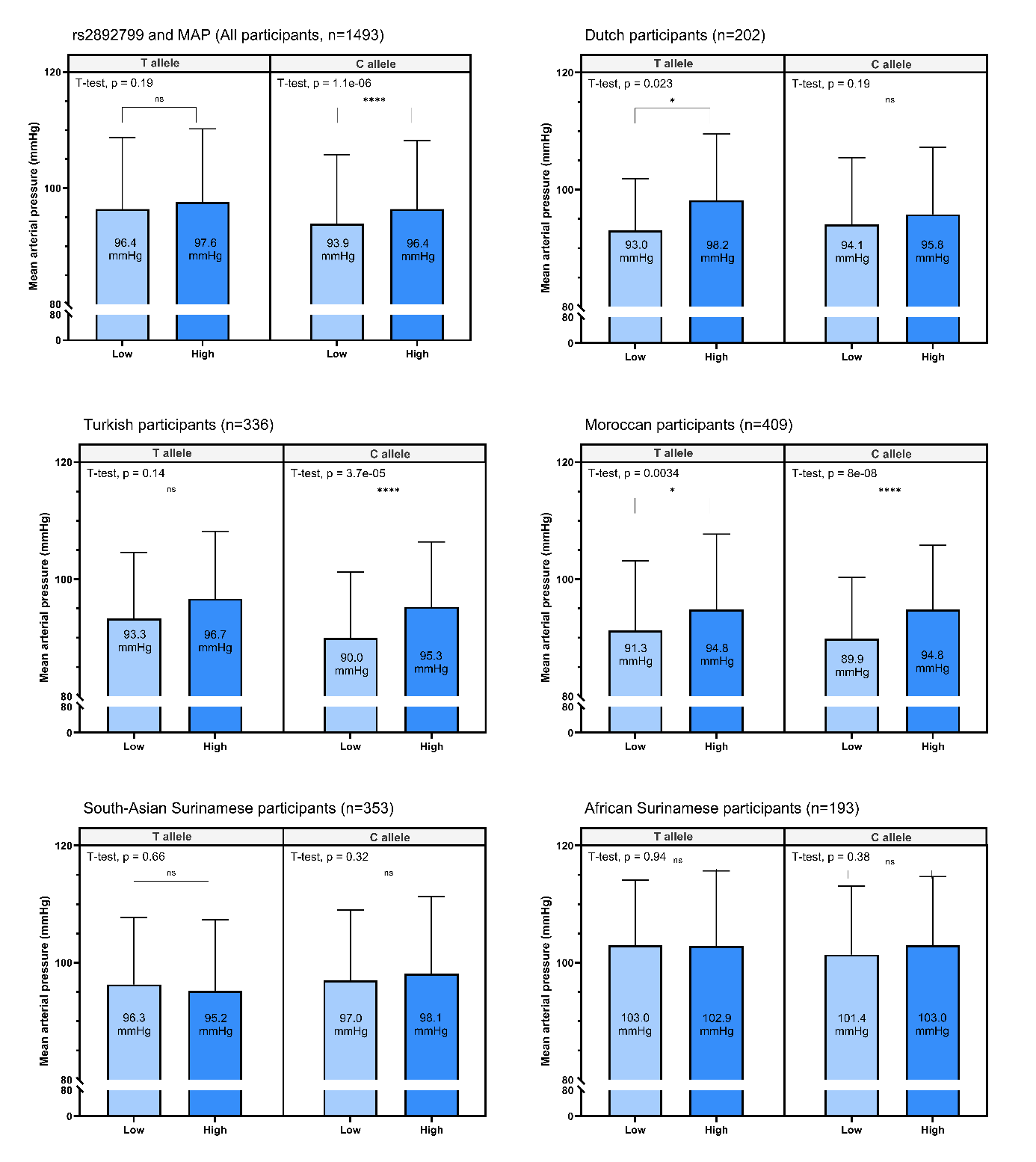
**

**Figure S7.** Mean arterial pressure for alleles of rs2892799 and sodium intake estimated with food frequency questionnaires for every ethnic group in the HELIUS population stratified for ethnicity.

**
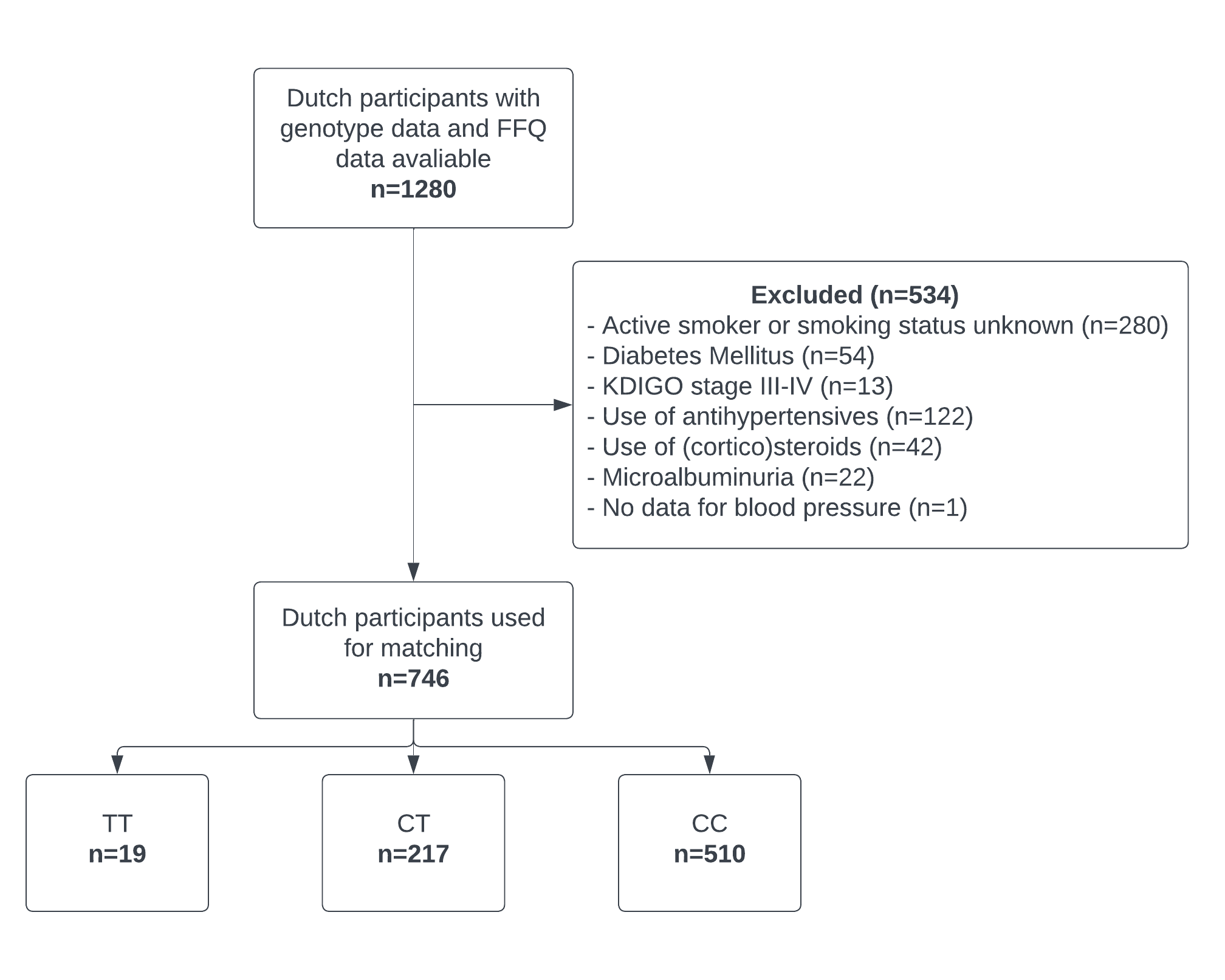
**

**Figure S8.** Flowchart for selection Dutch HELIUS participants based on genotype. FFQ, food frequency questionnaire. KDIGO, Kidney Disease: Improving Global Outcomes
